# Occurrence of Honey Bee (*Apis mellifera* L.) Pathogens in Wild Pollinators in Northern Italy

**DOI:** 10.3389/fcimb.2022.907489

**Published:** 2022-06-30

**Authors:** Giovanni Cilia, Simone Flaminio, Laura Zavatta, Rosa Ranalli, Marino Quaranta, Laura Bortolotti, Antonio Nanetti

**Affiliations:** CREA Research Centre for Agriculture and Environment, Bologna, Italy

**Keywords:** wild bees, pollinators, pathogen transmission, managed honey bees, honey bee pathogens, Hymenoptera, biodiversity, spillover

## Abstract

Diseases contribute to the decline of pollinator populations, which may be aggravated by the interspecific transmission of honey bee pests and pathogens. Flowers increase the risk of transmission, as they expose the pollinators to infections during the foraging activity. In this study, both the prevalence and abundance of 21 honey bee pathogens (11 viruses, 4 bacteria, 3 fungi, and 3 trypanosomatids) were assessed in the flower-visiting entomofauna sampled from March to September 2021 in seven sites in the two North-Italian regions, Emilia-Romagna and Piedmont. A total of 1,028 specimens were collected, identified, and analysed. Of the twenty-one pathogens that were searched for, only thirteen were detected. Altogether, the prevalence of the positive individuals reached 63.9%, with *Nosema ceranae*, deformed wing virus (DWV), and chronic bee paralysis virus (CBPV) as the most prevalent pathogens. In general, the pathogen abundance averaged 5.15 * 10^6^ copies, with CBPV, *N. ceranae*, and black queen cell virus (BQCV) as the most abundant pathogens, with 8.63, 1.58, and 0.48 * 10^7^ copies, respectively. All the detected viruses were found to be replicative. The sequence analysis indicated that the same genetic variant was circulating in a specific site or region, suggesting that interspecific transmission events among honey bees and wild pollinators are possible. Frequently, *N. ceranae* and DWV were found to co-infect the same individual. The circulation of honey bee pathogens in wild pollinators was never investigated before in Italy. Our study resulted in the unprecedented detection of 72 wild pollinator species as potential hosts of honey bee pathogens. Those results encourage the implementation of monitoring actions aiming to improve our understanding of the environmental implications of such interspecific transmission events, which is pivotal to embracing a One Health approach to pollinators’ welfare.

## Introduction

Pollination is a pivotal ecosystem service to both natural and agricultural environments. Its global economic value is estimated to be on the order of hundreds of billions of dollars per year (Porto et al., 2020). That adds to an invaluable intrinsic contribution to biodiversity (Senapathi et al., 2015). Thus, the decline of pollinator populations is receiving increased attention, with a focus on the role played by pesticides (O’Neal et al., 2018; Siviter et al., 2018; Hrynko et al., 2021), habitat fragmentation (Hung et al., 2021; Librán-Embid et al., 2021), climatic change (Schenk et al., 2018; Duchenne et al., 2020; Cane, 2021), and urbanisation (Fortel, 2014; Choate et al., 2018; Hofmann and Renner, 2020; Wenzel et al., 2020). While the occurrence of interspecific food competition events between wild species and managed honey bee (*Apis mellifera*) colonies are still debated (Tscharntke and Steffan-Dewenter, 2000; Wojcik et al., 2018; Rasmussen et al., 2021), little is known about the interspecific transmission of pathogens between honey bees and wild pollinators (Nanetti et al., 2021a). This last point is crucial, as the welfare of *A. mellifera* colonies depends on apicultural management, the associated economic value of apiculture (Ballantyne et al., 2017; Khalifa et al., 2021), and the equilibrium of the ecosystem in which honey bees live.

Interspecific pathogen transmission may occur with arthropods sharing the same environment as the honey bees. The main routes between established and new hosts include direct contact, orofecal exchanges, and the ingestion of pollen contaminated with pathogens (Singh et al., 2010; Cilia et al., 2021; Tehel et al., 2022). The infection may also occur during foraging *via* contact with pathogen-contaminated pollen, nectar, and floral organs (Chen et al., 2006a; Mazzei et al., 2014; Graystock et al., 2015; Alger et al., 2019b; Schittny et al., 2020). Wasps and hornets predating infected bees (Yañez et al., 2012; Forzan et al., 2017; Mazzei et al., 2018; Mazzei et al., 2019) and ants cannibalising their corpses (Sébastien et al., 2015; Cooling et al., 2017; Gruber et al., 2017) are likely to get infected. Interspecific transmission may affect also organisms that are not expected to come into direct contact with the honey bees, like spiders and beetles (Yue et al., 2007; Erler et al., 2012; Levitt et al., 2013).

Several studies have aimed at elucidating pathogen dynamics beyond interspecific transmission. Bees are considered the most efficient pollinators (Ballantyne et al., 2017). As many pollinators exploit the same floral resources as honey bees, horizontal transmission of pathogens, especially honey bee pathogens (Dalmon et al., 2021), becomes possible with other Hymenoptera species (Santamaria et al., 2018; Purkiss and Lach, 2019; Gusachenko et al., 2020; Ocepek et al., 2021), other pollinators (Bailes et al., 2018), and other arthropods (Nanetti et al., 2021a). Considering the importance of wild pollinators and the adaptive plasticity of pathogens transmitted *via* regular flower visits (Burnham et al., 2021; Cohen et al., 2022; Manley et al., 2019), our understanding of both ecosystem health and its impact on pollinator decline requires increased research on the interspecific interactions occurring in these ecosystems. The increasing number of studies reporting honey bee pathogens in other host species portrays a scenario consisting of one reservoir species and multiple spillover events.

Population studies might elucidate those aspects (Benjamin-Chung et al., 2018) that, in the case of wild bees, are complicated by those species’ peculiar biological and ecological characteristics (Koh et al., 2016; Drossart and Gérard, 2020; Prendergast et al., 2020). This makes spillover routes generally unknown and undetermined (Yañez et al., 2020).

This study was conducted within a nationwide Italian monitoring project on wild bees (BeeNet project). We aimed to investigate both the occurrence and circulation of the main honey bee pathogens in the wild pollinators of two North-Italian regions, contributing to assessing the risk of possible interspecific transmission and spillover.

## Material and Methods

### Sampling

**Figure 1:**
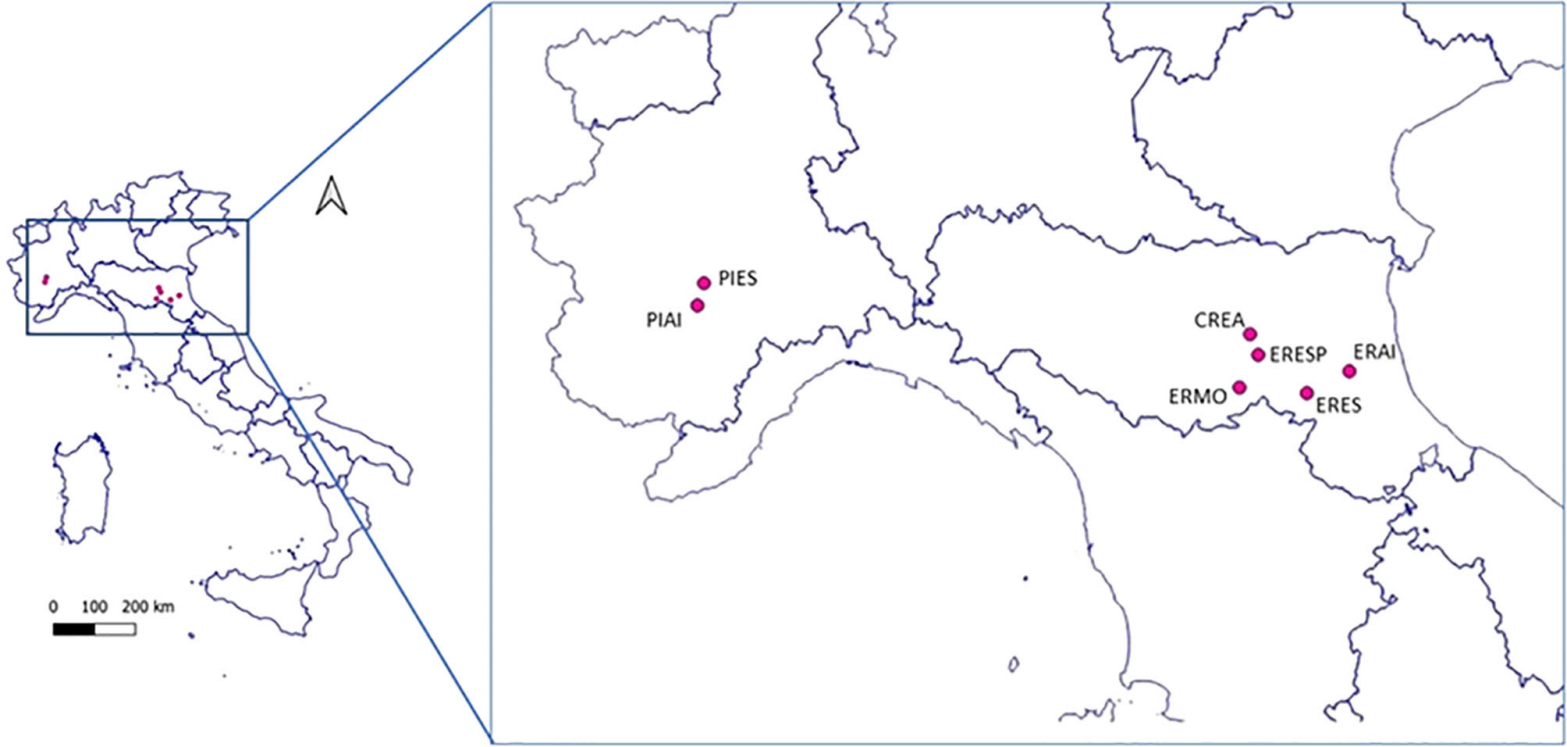
Geographical localisation of the investigated sampling sites.

Seven sites were chosen (**Figure 1**) for this study on the occurrence of honey bee pathogens in northern Italy. The geographical and environmental characteristics of each site are reported in **Table 1**. This study is part of a wider project to monitor wild bees, which aims to compare the communities of these pollinators in different agroecosystems; therefore, the locations were chosen *a priori* by the project. Two Land Cover Categories were identified based on the management of the agricultural areas: intensive (category 2.1.1—Agricultural areas, non-irrigated arable land) and semi-natural land (category 2.4.3—Heterogeneous agricultural areas, land principally occupied by agriculture, with significant areas of natural vegetation). Finally, a site in the urban area corresponding to the CREA-AA research centre was added and included in category 1.2.1.3 (Industrial, commercial, and transport units).

**Table 1:** Geographical and environmental characterisation of the sites included in the study.

Site	Agroecosystem	Region	City	Coordinates WGS84	CLC	a.s.l.
PIAI	Intensive	Piedmont	Cherasco (CN)	44°40′24.64″N; 7°48′44.93″E	2.1.1.1	293 m
PIES	Semi-natural	Piedmont	Zona di Salvaguardia dei Boschi e Rocche del Roero, Baroli (CN)	44°46′32.88″N; 7°51′10.84″E	2.4.3	346 m
CREA	Urban	Emilia-Romagna	Bologna (BO)	44°31′26.31”N; 11°21′3.23”E	1.2.1.3	36 m
ERAI	Intensive	Emilia-Romagna	Massa Castello (RA);	44°15′51.21”N; 12°8′13.52”E	2.1.1.1	14 m
ERMO	Semi-natural	Emilia-Romagna	Monzuno (BO)	44°16′51.05”N; 11°16′56.23”E	2.4.3	630 m
ERESP	Semi-natural	Emilia-Romagna	Parco Regionale dei Gessi Bolognesi e Calanchi dell’Abbadessa (BO)	44°25′39.08″N; 11°23′58.62″E	2.4.3	93 m
ERES	Semi-natural	Emilia-Romagna	Rocca San Casciano (FC)	44°05′00.52″N; 11°51′00.18″E	2.4.3	183 m

CLC, CORINE Land Cover category; a.s.l., above sea level.

Sampling sites were located in agricultural agroecosystems with different management practices; both intensively farmed areas and cultivated ones fragmented by natural elements were investigated. All sampling was carried out in field margins, or other landscape elements such as hedges and meadows, but always contiguous to cultivated areas.

Sampling was carried out once a month, from March to September 2021. At each site, an area with a high abundance of entomophilous plant species in anthesis was chosen to ensure that floral resources were sufficient and in high enough abundance to support a pollinator community with high species diversity. The sampling was conducted on sunny and non-windy days, with an average temperature above 15°C.

Sampling focused on wild bees, but honey bees, hoverflies, flies, wasps, and beetles were also collected when caught in the same sweep net action because they were on the same flowers as the targeted bees.

Pollinators were collected by one collector using the sweep net technique during one effective hour of sampling, stopping the timer at each catch. Each specimen was placed, depending on its size, in a sterile single 2-ml microtube or 15-ml tube. The tubes containing captured individuals were then placed in a cooler bag with freezer packs until arrival at the laboratory, where all the specimens were identified.

### Taxonomic Identification

On each sampling day, captured flower visitors were placed at −80°C for 30 min, after which they were identified to species level whenever possible, otherwise at the genus level. Identification was performed under a stereomicroscope, placing the individual in a Styrofoam container with dry ice to not degrade the RNA. After identification, samples were stored at −80°C until the analysis.

### Extraction of Nucleic Acids

Before extraction, all samples were washed with 95% ethanol to remove external microbial contaminations, and each sample was analysed individually. The sample was placed in a 2-ml microtube with 500 µl of DNA/RNA Shield (Zymo Research, Irvine, CA, USA) and crushed with a TissueLyser II (Qiagen, Hilden, Germany) for 3 min at 30 Hz, as previously reported (Cilia et al., 2019; Nanetti et al., 2021b). The obtained suspensions were split into two aliquots from which DNA and RNA were separately extracted.

The above-described procedures were accomplished by using a Quick DNA Microprep Plus Kit (Zymo Research) and Quick RNA Microprep Plus Kit (Zymo Research), respectively, following the modified manufacturer’s instructions for solid tissue processing (Mazzei et al., 2019; Nanetti et al., 2021c).

The obtained nucleic acids were eluted in 50 µl of DNAase-Rnase-free water, and the extracts were stored at −80°C until the qPCR assays.

### Real-Time Quantitative Assays to Detect DNA Pathogens

The extracted DNA was analysed using Real-Time PCR to quantify the abundance of detected bacteria and trypanosomatids in the samples, using the primers reported in **Table 2**. For each target, a total reaction volume of 15 µl was prepared as previously described (Cilia et al., 2020) using PowerUp™ SYBR™ Green Master Mix (Thermo Fisher, Waltham, MA, USA) with forward and reverse primers (2 µM) and 3 µl of DNA extract. The Real-Time PCR assay was performed on a QuantStudio™ 3 Real-Time PCR System (Thermo Fisher Scientific), following the protocols for all gene sequences (Dobbelaere et al., 2001; James and Skinner, 2005; Martin-Hernandez et al., 2007; Roetschi et al., 2008; Meeus et al., 2012; Huang et al., 2015; Arismendi et al., 2016; Cilia et al., 2018; Xu et al., 2018). DNA previously extracted from positive honey bee samples was used as positive controls. Sterile water was used as a negative control in all analytical steps. All the analyses were conducted in duplicate.

**Table 2:** List of primers used to detect fungi, bacteria, and trypanosomatids.

Target	Primer name	Sequence (5′–3′)	Reference
*Nosema ceranae*	Hsp70_F	GGGATTACAAGTGCTTAGAGTGATT	(Cilia et al., 2018)
	Hsp70_R	TGTCAAGCCCATAAGCAAGTG	
*Nosema apis*	321APIS_F	GGGGGCATGTCTTTGACGTACTATGTA	(Martin-Hernandez et al., 2007)
	321APIS_R	GGGGGGCGTTTAAAATGTGAAACAACTATG	
*Paenibacillus larvae*	AFB-F	CTTGTGTTTCTTTCGGGAGACGCCA	(Dobbelaere et al., 2001)
	AFB-R	TCTTAGAGTGCCCACCTCTGCG	
*Melissococcus plutonius*	MelissoF	CAGCTAGTCGGTTTGGTTCC	(Roetschi et al., 2008)
	MelissoR	TTGGCTGTAGATAGAATTGACAAT	
*Crithidia mellificae*	Cmel_Cyt_b_F	TAAATTCACTACCTCAAATTCAATAACATAATCAT	(Xu et al., 2018)
	Cmel_Cyt_b_R	ATTTATTGTTGTAATCGGTTTTATTGGATATGT	
*Lotmaria passim*	Lp2F 459	AGGGATATTTAAACCCATCGAA	(Arismendi et al., 2016)
	Lp2R 459	ACCACAAGAGTACGGAATGC	
*Crithidia bombi*	C.bombi_119Fw	CCAACGGTGAGCCGCATTCAGT	(Huang et al., 2015)
	C.bombi_119Rv	CGCGTGTCGCCCAGAACATTGA	
*Ascosphaera apis*	A_apis_3-F1	TGTCTGTGCGGCTAGGTG	(James and Skinner, 2005)
	A_apis_3-R1	CCACTAGAAGTAAATGATGGTTAGA	
*Spiroplasma apis*	FW As-636F	CGGGAGAATTTGTCCTATCG	(Meeus et al., 2012)
	REV As-636R	CCCACTTTAACAATCGGGATG	
*Spiroplasma melliferum*	FW Ms-160F	TTGCAAAAGCTGTTTTAGATGC	(Meeus et al., 2012)
	REV Ms-160R	TGACCAGAAATGTTTGCTGAA	

For each target, a standard curve was generated by amplifying serially diluted recombinant plasmids containing the pathogen-specific DNA fragment from 1 * 10^1^ to 1 * 10^9^ copies in a qPCR assay on a QuantStudio™ 3 Real-Time PCR System (Thermo Fisher Scientific), as previously reported (Cilia et al., 2020; Nanetti et al., 2021c), following the amplification and quantification protocols (Dobbelaere et al., 2001; Martin-Hernandez et al., 2007; Roetschi et al., 2008; Arismendi et al., 2016; Cilia et al., 2018; Xu et al., 2018).

### Real-Time Quantitative Assays to Detect Viral RNA

To quantify the virus abundance in the samples, all RNA extracts were analysed through Real-Time PCR using Power SYBR™ Green Cells-to-CT™ Kit (Thermo Fisher Scientific), as previously reported (Cilia et al., 2021). The primers used to amplify the target honey bee viruses considered here are reported in **Table 3**. The Real-Time PCR assay was performed on a QuantStudio™ 3 Real-Time PCR System (Thermo Fisher Scientific), following the protocols for each gene sequence. RNA previously extracted from positive honey bees was used as the positive control for each investigated virus. All the analyses were conducted in duplicates.

**Table 3:** List of primers used to detect viruses.

Target	Primer name	Sequence (5′–3′)	Reference
KBV	KBV 83F	ACCAGGAAGTATTCCCATGGTAAG	(Chantawannakul et al., 2006)
	KBV 161R	TGGAGCTATGGTTCCGTTCAG	
DWV	DWV Fw 8450	TGGCATGCCTTGTTCACCGT	(Mazzei et al., 2018)
	DWV Rev 8953	CGTGCAGCTCGATAGGATGCCA	
ABPV	APV 95F	TCCTATATCGACGACGAAAGACAA	(Chantawannakul et al., 2006)
	APV 159R	GCGCTTTAATTCCATCCAATTGA	
IAPV	IAPV B4S0427_R130M	RCRTCAGTCGTCTTCCAGGT	(Kajobe et al., 2010)
	IAPV B4S0427_L17M	CGAACTTGGTGACTTGARGG	
BQCV	BQCV 9195F	GGTGCGGGAGATGATATGGA	(Chantawannakul et al., 2006)
	BQCV 8265R	GCCGTCTGAGATGCATGAATAC	
SBV	SBV 311F 79	AAGTTGGAGGCGCGyAATTG	(Chantawannakul et al., 2006)
	SBV 380R	CAAATGTCTTCTTACdAGAGGyAAGGATTG	
CBPV	CPV 304F 79	TCTGGCTCTGTCTTCGCAAA	(Chantawannakul et al., 2006)
	CPV 371R	GATACCGTCGTCACCCTCATG	
SBPV	SPV 8383F 81	TGATTGGACTCGGCTTGCTA	(de Miranda et al., 2010)
	SPV 8456R	CAAAATTTGCATAATCCCCAGTT	
*Am*FV	AmFV2-F	ACCCAACCTTTTGCGAAGCGTT	(Hartmann et al., 2015)
	AmFV2-R	ATGGGGCGTCTCGGGTAACCA	
AIV	AIV12F	GGCTAGTAAACGTAGTGGATATGACAAT	(Chantawannakul et al., 2006)
	AIV106R	CACCTGGTGGTCCAAGAGAAG	
Moku virus	MKVqF	CTACAACGCACGCGAGTAGA	(Garigliany et al., 2017)
	MKVqR	CCTTTCAAAGCAACGCTACC	

KBV, Kashmir bee virus; DWV, deformed wing virus; ABPV, acute bee paralysis virus; IAPV, Israeli acute bee paralysis virus; BQCV, black queen cell virus; SBV, sac brood virus; CBPV, chronic bee paralysis virus; SBPV, slow paralysis virus; AmFV, Apis mellifera filamentous virus; AIV, Apis iridescent virus.

For each target, a standard curve was generated by amplifying the serially diluted recombinant plasmids containing the pathogen-specific RNA fragment from 1 * 10^1^ to 1 * 10^9^ copies in a qPCR assay on a QuantStudio™ 3 Real-Time PCR System (Thermo Fisher Scientific), as previously reported (Mazzei et al., 2019; Cilia et al., 2021), following the amplification and quantification protocols (Chantawannakul et al., 2006; de Miranda et al., 2010; Kajobe et al., 2010; Martin et al., 2012; Hartmann et al., 2015; Garigliany et al., 2017; Mazzei et al., 2018).

### Strand-Specific RT-PCR

The active replication of viruses was evaluated by performing strand-specific RT-PCRs using specific primers, as previously described (Mazzei et al., 2018; Nanetti et al., 2021b). Positive and negative strands previously obtained from positive honey bees were used as positive controls. The obtained cDNAs were amplified by PCR for the viral targets, and the amplicons were visualised on a 2% agarose gel. Subsequently, the amplicons were sequenced (BMR Genomics, Padua, Italy) and analysed using BLAST (Altschul et al., 1990).

A phylogenetical analysis was performed on each viral sequence deposited in GenBank using the maximum likelihood method and Tamura–Nei model (Tamura et al., 2004) (Saitou and Nei, 1987) associating taxa clustered together in the bootstrap test (500 replicates) (Felsenstein, 1985). Evolutionary analyses were conducted in MEGA X (Kumar et al., 2018).

### Statistical Analysis

Pathogen prevalence among months, sites, and pollinator taxon were analysed by a chi-square independence test. Since contingency tables contained values <5 and were wider than 2 × 2, Fisher’s exact test could not be applied. A simulated p-value was calculated based on 2,000 replicates.

The pathogen abundance was determined at the individual level by averaging the two technical replicates of each PCR assay. The results are reported in terms of average ± SD. The abundance of 13 pathogens in wild bees and other pollinators was also compared among months, sites, and taxa. Data were tested first for normality using the Shapiro–Wilk test. Since the normality check failed, data were analysed by a Kruskal–Wallis test, followed by a pairwise Wilcoxon test with Bonferroni correction as a *post-hoc* test. The significance threshold was set at p = 0.05. All the analyses were performed with R version 4.1.2 (R Core Team, 2021).

## Results

A total of 1,028 flower-visiting insects were captured and analysed altogether in the two considered Italian regions. The samples included the following: Apoidea (N = 835), non-Apoidea Hymenoptera (N = 68), Diptera (N = 107), and pollinators belonging to other taxa (N = 18) (**Table S1**). The bee specimens were recognised as follows: *Halictus* spp. (N = 166), *Lasioglossum* spp. (N = 142), *Bombus* spp. (N = 134), *Andrena* spp. (N = 121), *Megachile* spp. (N = 43), *Anthidium* spp. (N = 42), *Eucera* spp. (N = 34), *Osmia* spp. (N = 27), *Chelostoma* spp. (N = 20), *Ceratina* spp. (N = 19), *Hylaeus* spp. (N = 14), *Nomiapis diversipes* (N = 13), *Heriades* spp. (N = 12), *A. mellifera* (N = 12), *Lithurgus cornutus* (N = 6), *Xylocopa* spp. (N = 4), *Anthophora* spp. (N = 3), *Colletes* spp. (N = 3), *Melitturga clavicornis* (N = 3), *Stelis breviscula* (N = 3), *Systropha curvicornis* (N = 3), *Hoplitis* spp. (N = 2), *Habropoda tarsata* (N = 2), *Icteranthidium laterale* (N = 2), *Epeolus* spp. (N = 2), *Pseudoanthidium scapulare* (N = 1), *Sphecodes alternatus* (N = 1), and *Dasypoda hirtipes* (N = 1) (**Table S1**). The collected wasps and hornets were found to belong to the following: *Cerceris* spp. (N = 14), *Polistes* spp. (N = 12), and *Vespula* spp. (N = 4), plus a number of other specimens belonging to other genera (N = 28). The Diptera were classifies as *Syrphus* spp. (N = 16), *Epistrphus* spp. (N = 18), *Melanostoma* spp. (N = 13), *Sphaerophoria* spp. (N = 11), *Eristalis* spp. (N = 9), *Bombylius* spp. (N = 8), *Chloromyia* spp. (N = 7), *Villa* spp. (N = 4), and *Volucella* spp. (N = 3), plus a number other specimens belonging to other genera (N = 18) (**Table S1**).

### Pathogen Prevalence

**Figure 2:**
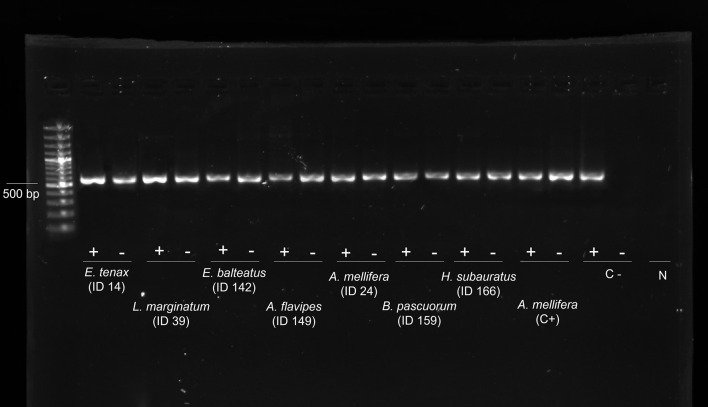
Evidence of genomic and replicative DWV strands. Gel electrophoresis of strand-specific RT-PCR of the cDNA from 8 individuals: genomic strand (+) and replicative strand (−). Positive control (C+): cDNA from replicative DWV of *Apis mellifera* workers. Negative control (C−): non-replicative DWV of *A. mellifera* workers. N: DNA- and Rnase-free water. DWV, deformed wing virus.

All the samples were negative for Israeli acute bee paralysis virus (IAPV), *Apis* iridescent virus (AIV), slow paralysis virus (SBPV), Moku virus, *Nosema apis*, *Crithidia mellificae*, *Paenibacillus larvae*, and *Melissococcus plutonius*. Except for the viruses mentioned above, all the other viruses were found to be present in the analysed samples in their replicative form (i.e., negative strand) (**Figure 2**).

A total of 712 of the sampled individuals were positive for at least one pathogen (69.3%). Altogether, *Nosema ceranae*, deformed wing virus (DWV), and chronic bee paralysis virus (CBPV) were the three most prevalent pathogens (16.1%, 15.7%, and 9.5%, respectively). Lower prevalence was observed for black queen cell virus (BQCV), acute bee paralysis virus (ABPV), sac brood virus (SBV), *Crithidia bombi*, *Spiroplasma melliferum*, *Spiroplasma apis*, *Am*FV, Kashmir bee virus (KBV), *Lotmaria passim*, and *Ascosphaera apis* (6.7%, 5.5%, 5.4%, 3.0%, 2.7%, 2.1%, 1.5%, 0.7%, 0.2%, and 0.2%, respectively) (**Table 4**).

**Table 4:** Prevalence of the positive samples per order or genus (ranked in alphabetical order) for the investigated pathogens (complete data are provided in **Table S1**).

Order/genus	No. samples	DWV		KBV		ABPV		CBPV		*Am*FV		SBV		BQCV		*Nosema ceranae*		*Lotmaria passim*		*Crithidia bombi*		*Spiroplasma apis*		*Spiroplasma melliferum*		*Ascosphaera apis*	
**Bees**																											
*Andrena* spp.	**121**	3.3%		9.9%		3.3%		0.8%		0		2.4%		22.3%		0		0		0		0		10.7%		0	
*Anthidium* spp.	**42**	21.4%		2.4%		11.9%		19.1%		2.4%		4.8%		4.8%		26.2%		0		2.4%		7.1%		2.4%		0	
*Anthophora* spp.	**3**	0		0		33.3%		0		0		0		0		0		0		0		0		0		0	
*Apis mellifera*	**12**	50.0%		0		16.7%		16.7%		0		8.3%		0		50.0%		0		0		0		0		0	
*Bombus* spp.	**134**	13.4%		0		4.8%		11.2%		1.5%		4.5%		26.9%		15.7%		0		20.9%		0		0.7%		0	
*Ceratina* spp.	**19**	15.8%		0		0		21.1%		0		5.3%		10.5%		5.3%		0		0		0		0		0	
*Chelostoma* spp.	**20**	50.0%		5.0%		0		0		0		0		0		5.0%		5.0%		0		0		0		0	
*Dasypoda* spp.	**1**	100%		0		0		0		0		0		0		0		0		0		0		0		0	
*Epeolus* spp.	**2**	0		0		0		0		0		0		0		100%		0		0		50.0%		0		0	
*Eucera* spp.	**34**	17.6%		0		2.9%		17.6%		0		14.7%		11.8%		11.8%		0		5.9%		2.9&		0		0	
*Halictus* spp.	**166**	18.7%		0		6.6%		13.9%		3.0%		7.8%		4.8%		21.1%		0		0		3.6%		4.2%		1.2%	
*Heriades* spp.	**12**	16.7%		0		0		25.0%		0		8.3%		0		8.3%		0		0		0		0		0	
*Hoplitis* spp.	**2**	50.0%		0		0		0		0		0		0		0		0		0		0		0		0	
*Hylaeus* spp.	**14**	21.4%		0		0		7.14%		0		7.14%		0		7.14%		0		0		0		0		0	
*Lasioglossum* spp.	**142**	11.3%		0.7%		2.1%		7.0%		0.7%		4.9%		5.6%		7.7%		0.7%		0		5.6%		2.1%		0	
*Lithurgus* spp.	**6**	0		0		16.7%		0		0		16.7%		16.7%		33.3%		0		0		0		0		0	
*Megachile* spp.	**43**	27.9%		0		7.0%		11.6%		2.3%		4.7%		2.3%		11.6%		0		0		4.7%		2.3		0	
*Melitturga* spp.	**3**	33.3%		0		0		33.3%		0		0		0		0		0		0		0		0		0	
*Nomiapis* spp.	**13**	15.4%		0		7.7%		15.4%		0		15.4%		7.7%		38.5%		0		0		0		0		0	
*Osmia* spp.	**27**	7.4%		0		7.4%		7.4%		0		18.5%		3.7%		11.1%		0		0		0		0		0	
*Pseudoanthidium* spp.	**1**	100%		0		0		0		0		100%		0		0		0		0		0		0		0	
*Stelis* spp.	**3**	33.3%		0		33.3%		0		0		0		0		0		0		0		0		0		0	
*Systropha* spp.	**3**	33.3%		0		0		0		0		0		0		0		0		0		0		0		0	
*Xylocopa* spp.	**4**	25.0%		0		0		25.0%		0		0		0		0		0		0		0		0		0	
**Wasp**																											
*Cerceris* spp.	**14**	25.7%		0		7.1%		0		0		7.1%		0		0		0		0		0		0		0	
*Polistes* spp.	**12**	16.7%		0		8.3%		16.7%		8.3%		25.0%		0		16.7%		0		0		0		0		0	
*Vespula* spp.	**4**	0		0		0		0		0		0		0		25.0%		0		0		0		0		0	
Other wasps	**35**	14.3%		0		5.7%		2.9%		0		5.7%		0		14.3%		0		0		0		0		0	
**Flies**																											
*Bombylius* spp.	**8**	0		0		0		0		0		12.5%		12.5%		12.5%		0		0		0		0		0	
*Episyrphus* spp.	**18**	30.8%		30.8%		7.7%		0		7.7%		0		7.7%		38.5%		0		0		0		0		0	
*Eristalis* spp.	**9**	22.2%		0		0		11.1%		0		0		0		66.7%		0		0		0		0		0	
*Melanostoma* spp.	**13**	23.1%		0		0		7.7%		0		0		0		0		0		0		0		7.7%		0	
*Syrphus* spp.	**16**	25.0%		0		0		0		6.3%		0		0		18.6%		0		0		0		0		0	
Other flies	**42**	16.7%		0		2.4%		14.3%		2.4%		2.4%		2.4%		9.5%		0		0		2.4%		2.4%		0	
**Other pollinators**																												
Hymenoptera	**11**	0		0		18.2		0		0		0		0		9.1%		0		0		0		0		0	
Coleoptera	**18**	11.1%		0		0		0		0		0		0		5.6%		0		0		0		0		0	
**Total**	**1,028**	**15.7%**		**0.7%**		**5.5%**		**9.5%**		**1.5%**		**5.4%**		**6.7%**		**16.1%**		**0.2%**		**3.0%**		**2.1%**		**2.7%**		**0.2%**	

In bold the total number of collected individual for each order/genus.

Considering all the sampling sites, *Chelostoma* spp. was found to be frequently infected by DWV (50.0%) and *L. passim* (5.0%), *Episyrphus* spp. by KBV (30.8%) and *Am*FV (7.7%), *Anthidium* spp. by ABPV (11.9%), *Heriades* spp. and *Ceratina* spp. by CBPV (25.0% and 21.1%, respectively), *Polistes* spp. by *Am*FV and SBV (8.3% and 25.0%), *Osmia* spp. by SBV (18.5%), *Bombus* spp. by BQCV (29.9%) and *C. bombi* (20.9%), *Andrena* spp. by BQCV (22.3%) and *S. melliferum* (10.7%), *Eristalis* spp. by *N. ceranae* (66.7%), and *Melanostoma* spp. by *S. melliferum* (7.7%) (**Table S2**).

The pathogen prevalence statistically differed with sampling site (χ^2^ = 246.35, p < 0.001), genus (χ^2^ = 615.48, p = 0.026), and month (χ^2^ = 237.81, p < 0.001).

### Pathogen Abundance

In general, CBPV was the most abundant pathogen (8.63 * 10^7^ ± 2.72 * 10^9^), followed by *N. ceranae* (1.58 * 10^7^ ± 3.56 * 10^8^), BQCV (4.84 * 10^6^ ± 1.52 * 10^8^), and DWV (8.40 * 10^5^ ± 1.54 * 10^7^) (**Table S3**). The same abundance trends were recorded in each region and site (**Table S3**).

The highest abundance was found in *Halictus scabiosae* for DWV (10 * 10^8^), in *Anthidium loti* and *Episyrphus balteatus* for KBV (10 * 10^5^); in *Halictus fulvipes*, *Andrena hattorfiana*, and *Osmia bicornis* for ABPV (10 * 10^6^); in *Eucera eucnemidea* for CBPV and SBV (10 * 10^10^ and 10 * 10^7^, respectively); in *Bombus sylvarum* for BQCV (10 * 10^9^); in *Anthidium florentinum* and *Lasioglossum villosulum* for *Am*FV (10 * 10^5^); in *H. fulvipes* and *Bombus terrestris* for *N. ceranae* (10 * 10^9^); in *Bombus pascuorum* and *B. terrestris* for *C. bombi* (10 * 10^7^); in *A. florentinum* and *E. eucnemidea* for *S. apis* (10 * 10^5^); and in *Halictus simplex* and *Andrena distinguenda* for *S. melliferum* (10 * 10^6^) (**Table S1**). Two individuals (*Chelostoma rapunculi* and *Lasioglossum malachurum*) sampled in July in ERMO were positive for *L. passim* (10 * 10^3^) (**Table S1**), while two individuals sampled in July, *H. simplex* and *Halictus cochlearitarsis*, respectively, from PIAI and ERES were found positive for *A. apis* (10 * 10^5^) (**Table S1**).

For all pathogens except CBPV, *L. passim*, and *S. apis*, the host taxon, sampling site, and month were significant predictors of abundance (**Table 5**). All three factors mentioned above were significantly correlated with the abundance of DWV, BQCV, *N. ceranae*, and *C. bombi*.

**Table 5:** Significativity of the sampling site, taxon, and month of the collection as predictors of pathogen abundance (Kruskal–Wallis analysis).

Pathogen	Site	Taxon	Month
**DWV**	Chi-squared = 29.92, df = 6, **p < 0.001**	Chi-squared = 79.289, df = 34, **p < 0.001**	Chi-squared = 31.83, df = 7, **p < 0.001**
**KBV**	Chi-squared = 41.68, df = 6, **p < 0.001**	Chi-squared = 121.58, df = 34, **p < 0.001**	Chi-squared = 11.613, df = 7, p > 0.05
**ABPV**	Chi-squared = 17.112, df = 6, **p = 0.008**	Chi-squared = 32.524, df = 34, p > 0.05	Chi-squared = 27.903, df = 7 **p < 0.001**
**CBPV**	Chi-squared = 6.6937, df = 6, p > 0.05	Chi-squared = 38.232, df = 34, p > 0.05	Chi-squared = 11.416, df = 7, p > 0.05
***Am*FV**	Chi-squared = 11.208, df = 6, p > 0.05	Chi-squared = 15.536, df = 34, p > 0.05	Chi-squared = 20.12, df = 7, **p = 0.005**
**SBV**	Chi-squared = 12.328, df = 6, p > 0.05	Chi-squared = 59.843, df = 34, **p < 0.001**	Chi-squared = 35.073, df = 7, **p < 0.001**
**BQCV**	Chi-squared = 41.245, df = 6, **p < 0.001**	Chi-squared = 98.875, df = 34, **p < 0.001**	Chi-squared = 39.953, df = 7, **p < 0.001**
***Lotmaria passim***	Chi-squared = 7.7031, df = 6, p > 0.05	Chi-squared = 24.337, df = 34, p > 0.05	Chi-squared = 4.9827, df = 7, p > 0.05
***Nosema ceranae***	Chi-squared = 26.165, df = 6, **p < 0.001**	Chi-squared = 77.389, df = 34, **p < 0.001**	Chi-squared = 28.383, df = 7, **p < 0.001**
***Crithidia bombi***	Chi-squared = 40.988, df = 6, **p < 0.001**	Chi-squared = 152.09, df = 34, **p < 0.001**	Chi-squared = 37.703, df = 7, **p < 0.001**
***Spiroplasma apis***	Chi-squared = 11.47, df = 6, p > 0.05	Chi-squared = 45.525, df = 34, p > 0.05	Chi-squared = 9.0193, df = 7, p > 0.05
***Spiroplasma melliferum***	Chi-squared = 21.919, df = 6, **p < 0.001**	Chi-squared = 38.255, df = 34, p > 0.05	Chi-squared = 29.07, df = 7, **p < 0.001**
***Ascosphaera apis***	Chi-squared = 13.894, df = 6, **p = 0.03**	Chi-squared = 9.1063, df = 34, p > 0.05	Chi-squared = 4.9827, df = 7, p > 0.05

Significant values are shown in bold.

Virus abundance is shown in **Figure S1**. The results of a *post-hoc* analysis are reported in **Table S4**. Briefly, the abundance of DWV was significantly higher in *A. mellifera*, *Megachile* spp. *Chelostoma* spp., *Dasypoda* spp., and *Pseudoanthidium* spp. compared to *Andrena* spp. (p < 0.005). PIES and PIAI reported the highest DWV abundance compared to ERESP (p < 0.005). *Episirphus* spp. showed higher KBV abundance compared to *Halictus* spp., *Bombus* spp., and *Andrena* spp. (p < 0.000), whereas KBV abundance was higher in PIAI compared to ERES, CREA, and PIES (p < 0.005). ABPV abundance was higher in ERAI related to ERESP (p < 0.001). SBV abundance was significantly lower in *Andrena* spp. compared to *Pseudoanthidium* spp., *Polistes* spp., *Osmia* spp., *Lithurgus* spp., and *Nomiapis* spp. (p < 0.01). BQCV was significantly higher in *Bombus* spp. than in *Andrena* spp., *Halictus* spp., and *Lasioglossum* spp. (p < 0.005), whereas in ERESP, a lower BQCV abundance was detected compared to ERAI and ERMO (p < 0.05). Host genus, sampling site, and month were not significant predictors of CBPV and *Am*FV abundance (p > 0.05).

The abundance of DNA pathogens is shown in **Figure S2**. The results of a *post-hoc* analysis are reported in **Table S4**. Briefly, a higher *N. ceranae* abundance was recorded in *Eristalis* spp. compared to *Lasioglossum* spp. and *A. mellifera* (p < 0.005), while in PIES, its abundance was higher than that in ERESP and ERMO (p < 0.05). The abundance of *C. bombi* was higher in *Bombus* spp. than *Halictus* spp., *Lasioglossum* spp., and *Andrena* spp. (p < 0.001), with significantly higher values in ERMO compared to ERES and PIES (p < 0.005). *S. melliferum* was significantly less abundant in ERMO than in ERESP (p < 0.005). Host genus, sampling site, and month were not significant predictors of *L. passim* and *S. apis* abundance (p > 0.05).

### Seasonal Trends

**Figure 3:**
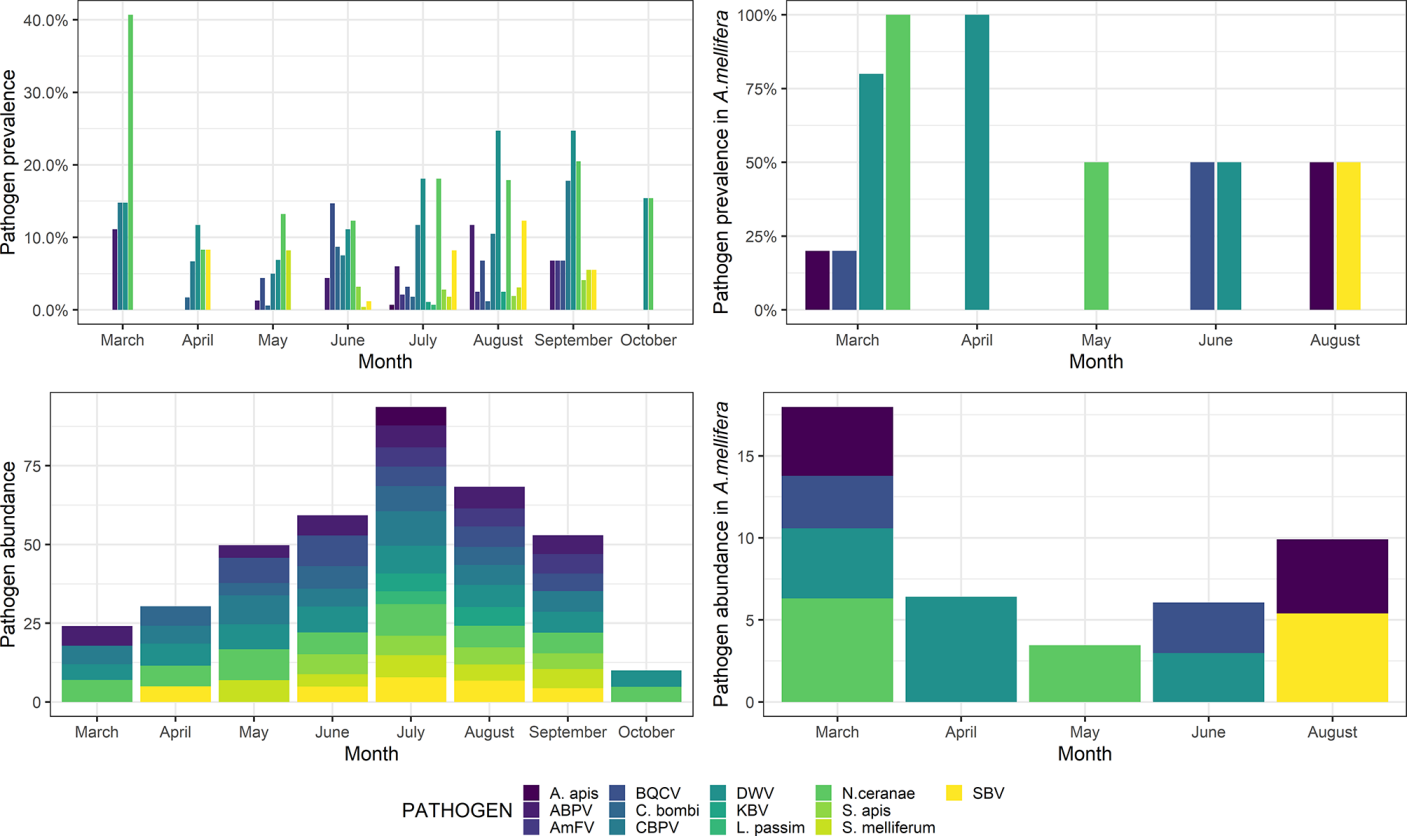
Pathogen prevalence (top) and abundance (bottom) throughout the sampling season (March–October) in wild species (left) and *Apis mellifera* (right). Abundance is shown as a decimal logarithm.

In March, a high prevalence of pathogens was detected, mainly due to the frequent occurrence of both *N. ceranae* and DWV. After a steep decrease, both *N. ceranae* and DWV resumed increasing, reaching a peak in September. However, the overall pathogen abundance peaked in July, after a steady increase during the previous seasons (**Figure 3**).

Detailed *post-hoc* comparisons are reported in **Table S4**. Statistical differences in pathogen abundance are shown in **Figures S1**, **S2**. Briefly, in March, a significantly higher abundance of ABPV and *N. ceranae* was detected, whereas the abundance of *S. melliferum* was significantly higher in May. Also, the abundance of BQCV and *C. bombi* was significantly higher in June, the abundance of DWV and SBV was significantly higher in August, and the abundance of *Am*FV was significantly higher in September. No significant differences were observed for KBV, CBPV, *L. passim*, *S. apis*, and *A. apis* (p > 0.05).

### Virus Phylogenesis

The viral sequences were studied to elucidate possible structural spatial and/or temporal similarities. All the sequenced viruses isolated from the different pollinator species belonged to the same strains. Comparing the positive individuals for each viral sequence resulted in perfect homology. Therefore, the subsequent analyses were conducted at the level of the host species rather than individually. All the viral sequences were similar to the European virus sequences available in GenBank.

**Figure 4:**
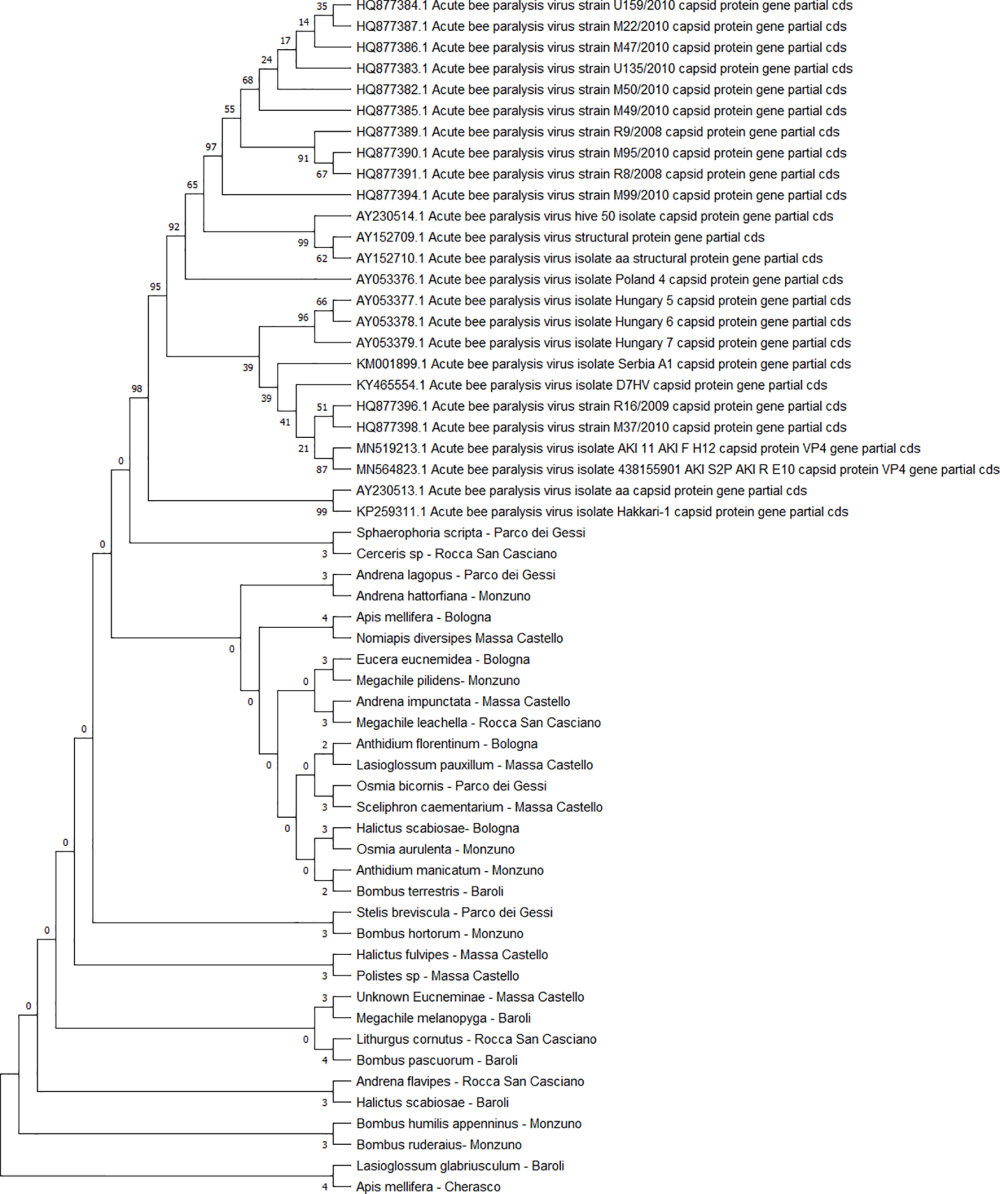
Maximum likelihood nucleotide phylogeny of ABPV *capsid protein* gene. This analysis involved 57 nucleotide sequences. There were a total of 879 positions in the final dataset. Only those with bootstrap > 50% are reported. Species and sampling sites are reported for the sequences identified in this study. ABPV, acute bee paralysis virus.

**Figure 5:**
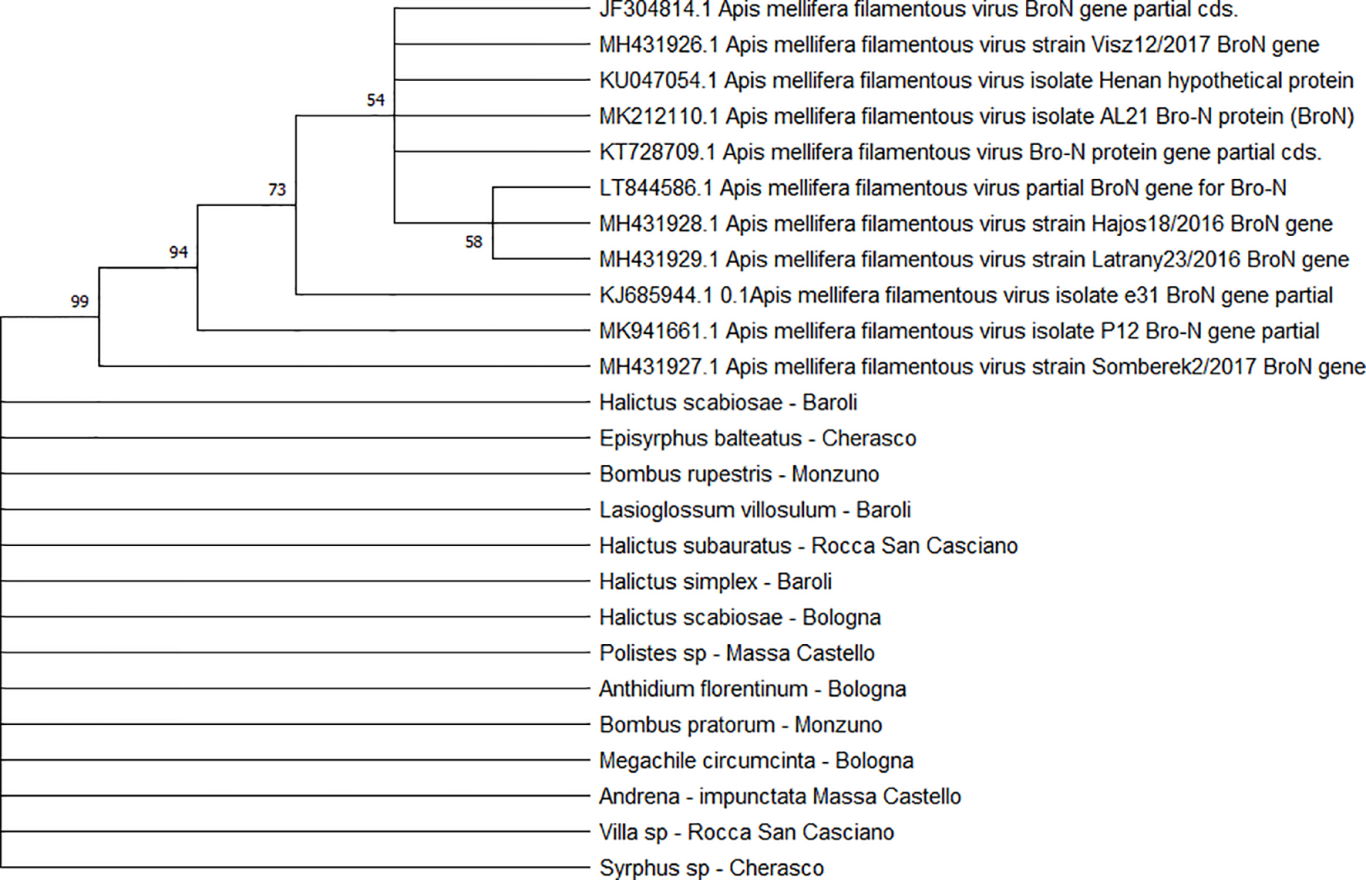
Maximum likelihood nucleotide phylogeny of *Am*FV *BroN* gene. This analysis involved 25 nucleotide sequences. There were a total of 896 positions in the final dataset. Only those with bootstrap > 50% are reported. Species and sampling sites are reported for the sequences identified in this study.

All the sequences belonging to ABPV (n = 33) (**Figure 4**) and *Am*FV (n = 14) (**Figure 5**) that were recognised in this study clustered in a large single clade. The sequences did not differ among the various host species and sampling sites.

**Figure 6:**
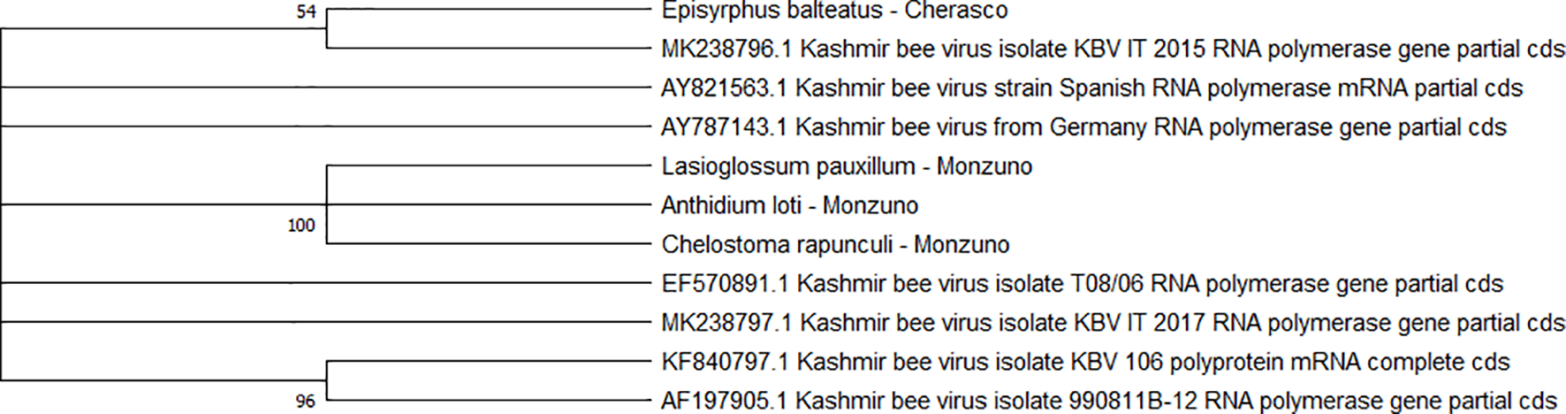
Maximum likelihood nucleotide phylogeny of KBV *RNA polymerase* gene. This analysis involved 11 nucleotide sequences. There were a total of 421 positions in the final dataset. Only those with bootstrap > 50% are reported. Species and sampling sites are reported for the sequences identified in this study. KBV, Kashmir bee virus.

Two separate clades were identified for the KBV sequences (n = 4). One of them was associated with three isolates from ERMO sites and the remaining with one PIAI site (**Figure 6**).

**Figure 7:**
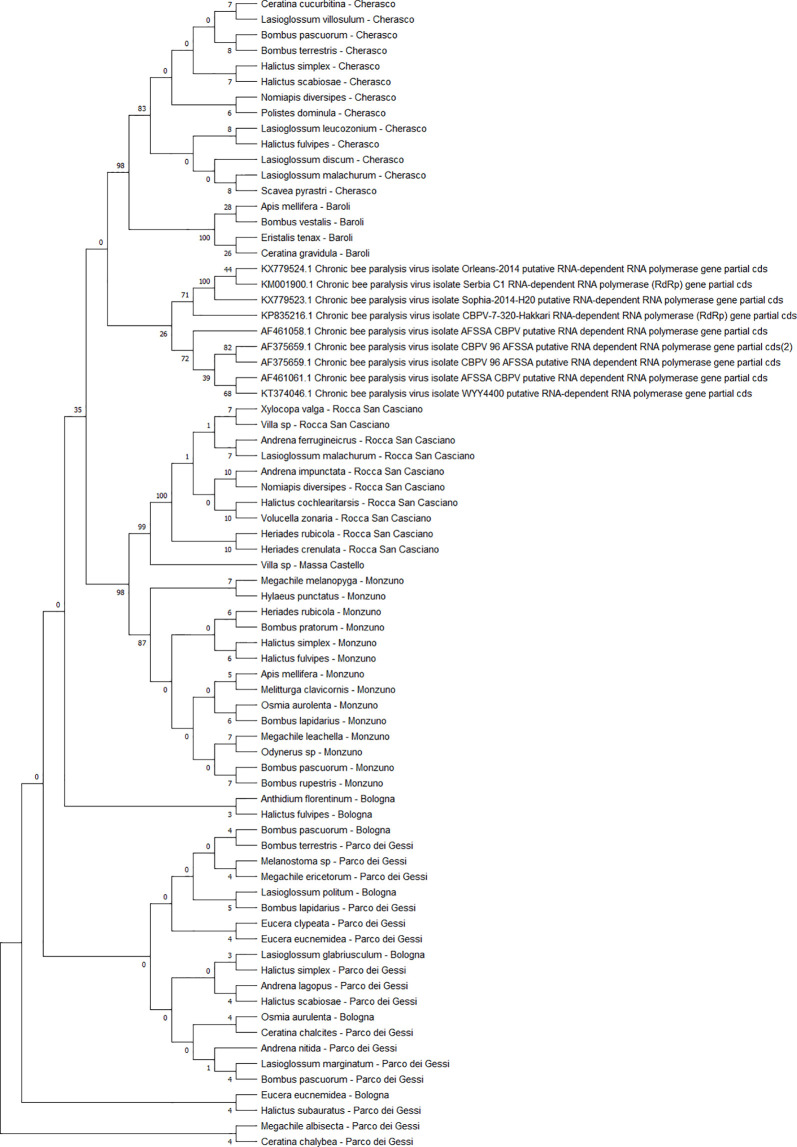
Maximum likelihood nucleotide phylogeny of CBPV *RNA-dependent RNA-polymerase* gene. This analysis involved 74 nucleotide sequences. There were a total of 583 positions in the final dataset. Only those with bootstrap > 50% are reported. Species and sampling sites are reported for the sequences identified in this study. CBPV, chronic bee paralysis virus.

**Figure 8:**
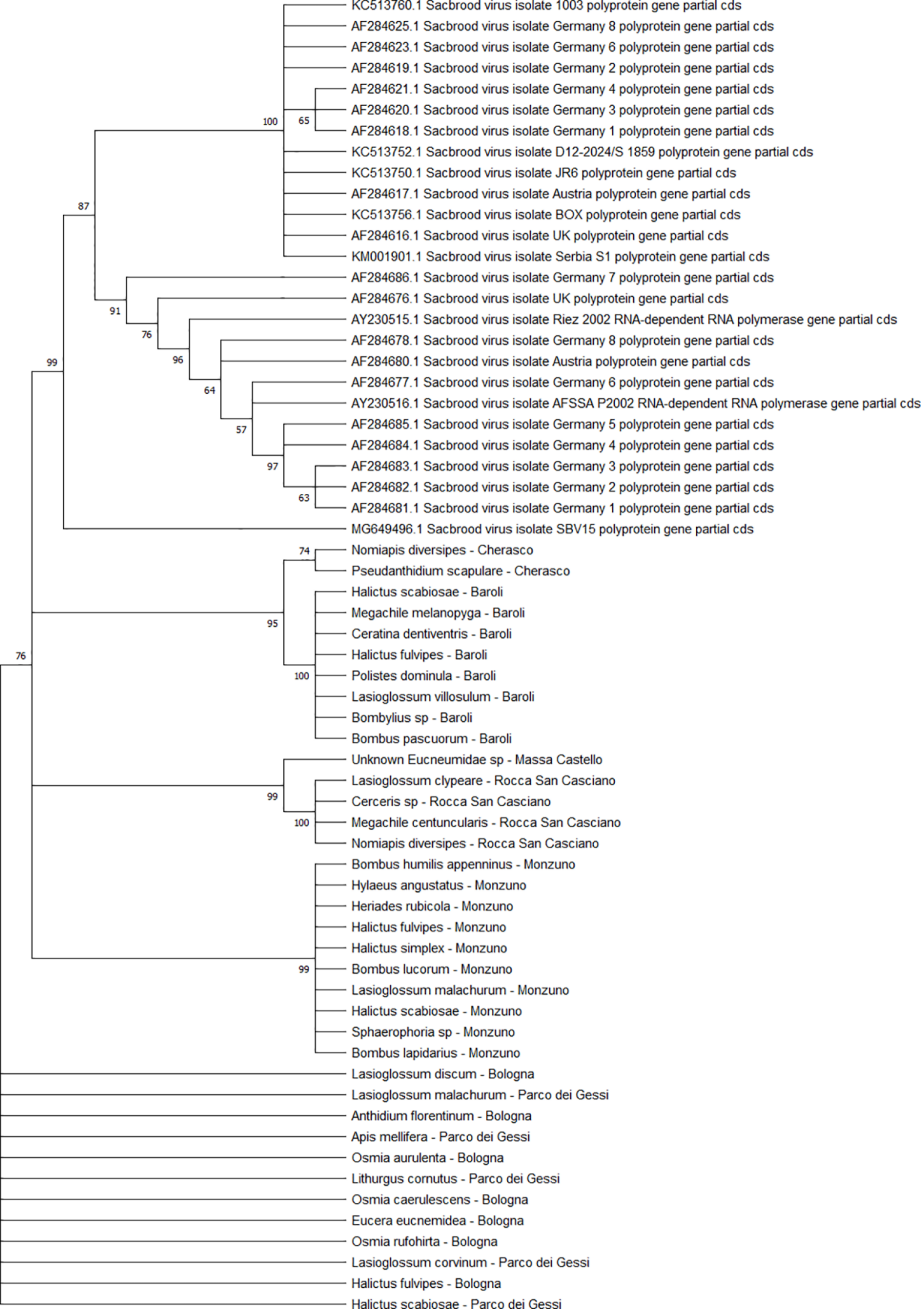
Maximum likelihood nucleotide phylogeny of SBV *polyprotein* gene. This analysis involved 63 nucleotide sequences. There were a total of 581 positions in the final dataset. Only those with bootstrap > 50% are reported. Species and sampling sites are reported for the sequences identified in this study. SBV, sac brood virus.

The detected CBPV sequences (n = 65) (**Figure 7**) and SBV sequences (n = 37) (**Figure 8**) clustered in 6 clades each, which were associated with different sampling sites. Both CBPV and SBV sequences had complete homology for the CREA and ERESP isolates.

**Figure 9:**
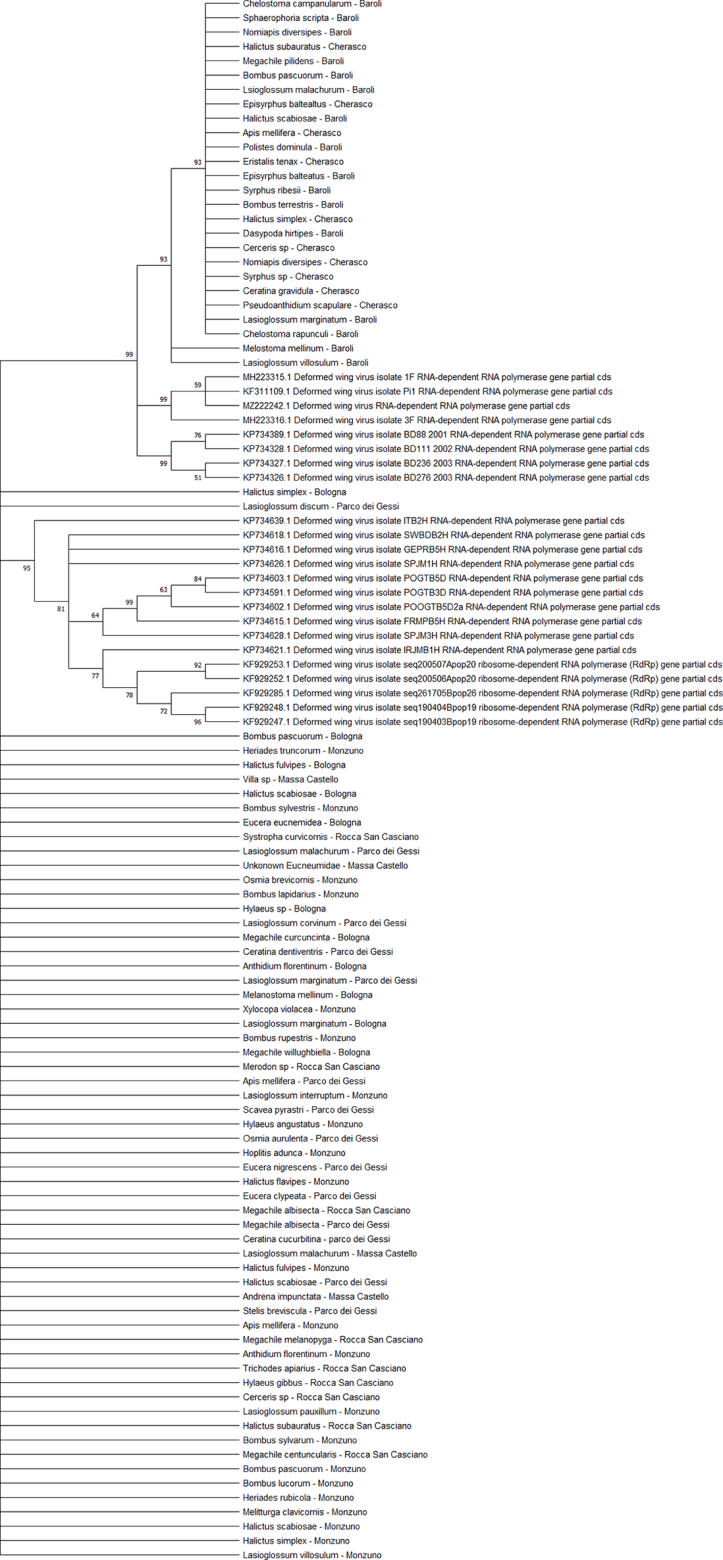
Maximum likelihood nucleotide phylogeny of DWV-A *RNA-dependent RNA-polymerase* gene. This involved 109 nucleotide sequences. There were a total of 562 positions in the final dataset. Only those with bootstrap > 50% are reported. Species and sampling sites are reported for the sequences identified in this study. DWV, deformed wing virus.

All DWV sequences (n = 86) belonged to the DWV-A strain, which is the most frequent and the least virulent variant strain in honey bees, and split into two regional clades (Emilia-Romagna and Piedmont) (**Figure 9**). Exceptions to this observation were two sequences from PIES (*Melanostoma mellinum* and *L. villosulum*; bootstrap = 93%), *H. simplex* from CREA, and *Lasioglossum discum* from ERESP (bootstrap = 97%).

**Figure 10:**
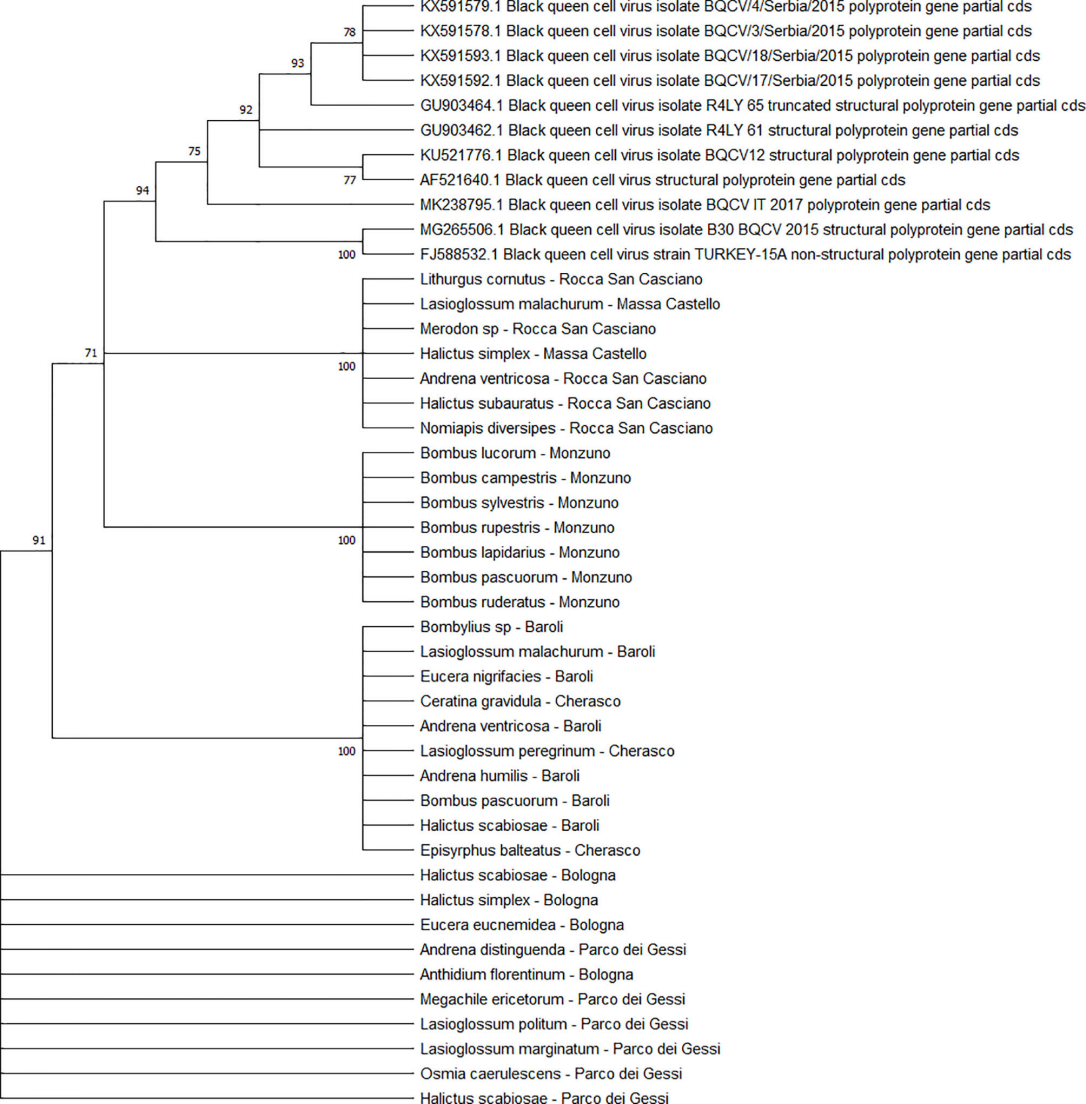
Maximum likelihood nucleotide phylogeny of BQCV *polyprotein* gene. This analysis involved 45 nucleotide sequences. There were a total of 701 positions in the final dataset. Only those with bootstrap > 50% are reported. Species and sampling sites are reported for the sequences identified in this study. BQCV, black queen cell virus.

The BQCV sequences (n = 34) clustered in 4 different clades (**Figure 10**), including the isolates from ERES and ERAI, the sites in the Piedmont region, CREA and ERESP, and ERMO.

### Co-Infections

Forty-five sampled specimens were found to be co-infected with two or more pathogens. The most complex co-infections were detected in two *B. pascuorum* individuals that were sampled in PIES. One of them was positive for DWV, SBV, *N. ceranae*, and *C. bombi*, and the other was positive for CBPV, SBV, BQCV, and *N. ceranae* (**Table S1**).

**Figure 11:**
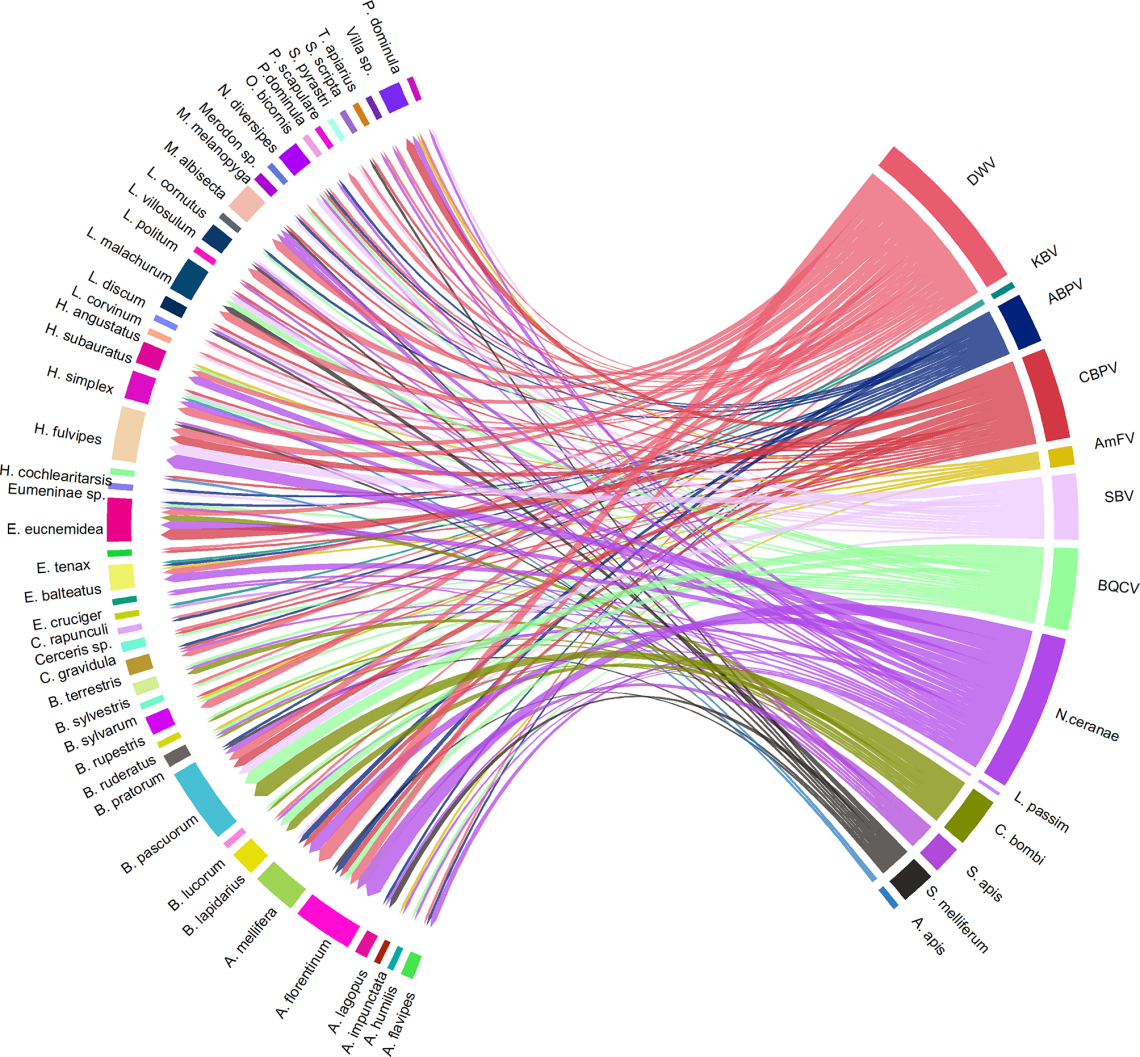
A visual schematisation of the subsample of pathogens that were involved in multiple infections related to their hosts. The arrow thickness denotes the number of co-infections observed within the same host species.

As reported in **Figure 11**, the most frequent co-infections included *N. ceranae*, DWV, CBPV, SBV, and BQCV. *Bombus* spp. and *Halictus* spp. were the two genera in which the highest number of multiple infections was found.

## Discussion

This study was conducted in Italy and confirms that interspecific transmission of pathogens between honey bees and wild pollinators may occur, as reported previously for other countries (Graystock et al., 2013b; Ravoet et al., 2014; Ngor et al., 2020; Pritchard et al., 2021). To the best of our knowledge, this topic has never before been approached in Italian surveys.

**Table S6** shows the unprecedented detection of honey bee pathogens in 72 wild bee species, which have shown the potential to act as alternative hosts (Nanetti et al., 2021a). These findings confirm that the considered pathogens show sufficient plasticity to adapt to multiple hosts in nature and the potential to impact the ecosystems involved. The viruses considered were found to be replicative in positive individuals, confirming that their adaptability to alternative host species may result in active infections (Celle et al., 2008; Bailes et al., 2018).

However, our understanding of the pathogenicity of those microorganisms in the alternative hosts is far from complete. Unlike the wing deformations associated with DWV infections in bumblebees, *Vespa crabro* and *Vespa velutina* (Genersch et al., 2006; Forzan et al., 2017; Dalmon et al., 2019a; Cilia et al., 2021), the examination of these insects sampled in this study did not show known clinical symptoms that could be ascribed to the pathogens under consideration. This observation is not astounding, as previous attempts to artificially infect bumblebee individuals and other wild bees have resulted in asymptomatic infections (Gusachenko et al., 2020). Nevertheless, artificial inoculations with viruses of the AKI complex group (ABPV, KBV, and IAPV) have reduced the reproductive success of bumblebees and increased the mortality rate in the populations studied (Meeus et al., 2014; Piot et al., 2015). For the time being, the available picture of symptomatic, asymptomatic, and subclinical infections makes it challenging to assess the real impact of honey bee pathogens on the individual wild pollinator species and their communities.

Overall, 69.3% of the individuals sampled in our study were positive for at least one pathogen. That proportion is in line with the prevalence observed in studies conducted in the United States (66% and 80.4%) (Levitt et al., 2013; Dolezal et al., 2016) and in France (79%) where overestimation may have occurred due to pooled sample analysis (Dalmon et al., 2021).

The pathogens that were considered in our study have been reported as transmissible by flowers and pollen (Alger et al., 2019a; Yañez et al., 2020; Nanetti et al., 2021a). Pathogens born by infected foragers (namely, *A. mellifera*) may persist on flowers, where susceptible pollinators of other species may be infected, with risk dependent on the number of floral visits (Graystock et al., 2013a; Mazzei et al., 2014; Alger et al., 2019b; Burnham et al., 2021).

In this study, DWV, *N. ceranae*, and CBPV were the pathogens detected with the highest frequency. Likewise, both DWV and microsporidia have been found in a broad range of pollinators (Nanetti et al., 2021a), which implies adaptability to the alternative hosts. As reported in other non-*Apis* hosts (Forzan et al., 2017; Mazzei et al., 2018; Cilia et al., 2021; Nanetti et al., 2021b), all the detected DWV sequences were type A, which is known as a common and weakly virulent genetic variant in honey bees (McMahon et al., 2016; Mordecai et al., 2016). The frequent occurrence of CBPV could be related to an increased prevalence of this virus in honey bees, which justifies concerns about its agricultural and environmental consequences (Vargas et al., 2017; Budge et al., 2020).

We consider of particular interest our results on *L. passim* and KBV. Presently, scant information about the spread of trypanosomatid infections in bees is available in Italy. However, in northern Italy, *L. passim* has been recently found both in apiary conditions of the Veneto region (Bordin et al., 2022) and as e-DNA in honey samples from the Emilia-Romagna region (Ribani et al., 2021). The epidemiological picture is still incomplete; nonetheless, the detection of *L. passim* in two of our wild bee samples collected in ERMO corroborates the effective circulation of this trypanosomatid in the environment. Similarly, the limited Italian reports about KBV refer to central regions (one case in the Tuscany region and two in the Latium region) (Cersini et al., 2013). Active infections in our wild bee samples from ERMO and PIAI suggest a broader spread of this honey bee virus. This finding recommends the use of non-*Apis* species as potential “sentinels” providing early epidemiological information about the environmental KBV presence.

The average pathogen abundance measured in the wild species considered in this study was lower than the threshold (>1 * 10^6^ copies) generally considered necessary for symptomatic infection in honey bees (Chen et al., 2006b; Mazzei et al., 2014; Martín-Hernández et al., 2018; Cilia et al., 2020), though various individuals exceeded this limit, and some of them reached 1 * 10^10^ genic copies. However, abundance does not provide definitive epidemiological information, as the symptomatic threshold is unknown yet for most pathogens and host species.

In general, the prevalence of the pathogens in this study showed a seasonal peak in March, followed by a quick decrease and a subsequent gradual increase until late summer. The cumulative abundance had a different trend, as it gradually increased monthly to peak in July when a decrease proceeded to reach a minimum in October. These results are in line with the seasonal pattern the same pathogens show in the *A. mellifera* colonies, where they often peak in spring/summer with a possible return in the late season (Tentcheva et al., 2004; D’Alvise et al., 2019; Dalmon et al., 2019b; Loeza-Concha et al., 2020; Chen et al., 2021).

In March, the comparatively high prevalence of positive samples was influenced by the frequent cases of *N. ceranae.* This finding agrees with the infection dynamics often occurring in the honey bees, where that pathogen may have an acute development in early spring depending on the colony development (Ptaszyńska et al., 2021; Ugolini et al., 2021; Formato et al., 2022). The subsequent decrease in nosemosis suggests a dilution due to the increased species richness, as previously reported for some viruses (Fearon and Tibbetts, 2021); however, a similar dilution effect was not common to all pathogens. The highest richness of pathogens corresponded to the peak in abundance recorded in July. That increase may be associated with both the development of the honey bee colonies and the availability of floral resources. Indeed, after the winter, the colony starts the growth of its population, which is intended to peak in summer, with an increased probability both of intra-colonial (Smith et al., 2013; Steinhauer et al., 2018; D’Alvise et al., 2019; Chen et al., 2021) and interspecific environmental transmission (Bartlett et al., 2019; Wilfert, 2021). Furthermore, the reduced flower availability in summer prompts pollinators to concentrate on the limited resources available, increasing the probability of pathogen exchanges among the potential host species.

Finally, our study showed repeated cases of co-infection in the wild bee species, as already reported for *A. mellifera* and *Aethina tumida* (Hung et al., 1995; Evans and Schwarz, 2011; Meeus et al., 2014; Nanetti et al., 2021b). Multiple infections are common in natural environments; nonetheless, we have a limited understanding of the interactions occurring among the involved pathogens (Armitage et al., 2022). Often, multiple infections reduce pathogen virulence due to antagonism (Garbutt et al., 2011), but in other cases, synergistic interactions may increase virulence and reduce the host lifespan (Clay and Rudolf, 2019; Armitage et al., 2022).

## Conclusion

This study demonstrates the environmental circulation of honey bee pathogens in the wild pollinating entomofauna present in two different North-Italian regions. The presence of the replicative forms of viruses affecting the honey bees suggests effective interspecific transmission between *A. mellifera* and alternate host species. To elucidate the effects of those infections on wild pollinators, studies are needed on their fitness, behaviour, mating and reproductive success, nesting, pollen stores, and larval development. Spillover of honey bee pathogens may have undetected yet important drawbacks to the health and functioning of an ecosystem. Health management of honey bee colonies is of high importance in this context, and the beekeepers should therefore undertake the consequent responsibility of being an essential component of the One Health concept (Wilfert et al., 2021).

## Data Availability Statement

The datasets presented in this study can be found in online repositories. The names of the repository/repositories and accession number(s) can be found in the article/**Supplementary Material**. Sequences were submitted to Genbank under the accession numbers ON304218 to ON304221 (KBV); ON304222 to ON304252 (ABPV); ON448627 to ON448640 (AmFV); ON448642 to ON448727 (DWV); ON448728 to ON448764 (SBV); ON448765 to ON448798 (BQCV): ON448799 to ON448863 (CBPV).

## Author Contributions

The study was designed by GC and LB. Samples were collected by GC, SF, LZ, and RR. The insect specimens were identified by SF, and the laboratory analyses were performed by GC. Data were analysed by GC, SF, LZ, RR, AN, and LB. GC, SF, LZ, and RR wrote the first version of the manuscript, which was revised by all the authors (MQ, AN, and LB). LB managed the project and the funding acquisition. All authors listed have made a substantial, direct, and intellectual contribution to the work and approved it for publication.

## Funding

This study was supported by the project BeeNet (Italian National Fund under FEASR 2014–2020) from the Italian Ministry of Agricultural, Food and Forestry Policies (MIPAAF).

## Conflict of Interest

The authors declare that the research was conducted in the absence of any commercial or financial relationships that could be construed as a potential conflict of interest.

## Publisher’s Note

All claims expressed in this article are solely those of the authors and do not necessarily represent those of their affiliated organizations, or those of the publisher, the editors and the reviewers. Any product that may be evaluated in this article, or claim that may be made by its manufacturer, is not guaranteed or endorsed by the publisher.

## Supplementary Material

Supplementary Figure 1 Virus abundance related to the site, month, and genus. Data are presented log10 transformation for better visualization. Means are visualized are red dots. Different number of asterisks indicate statistical differences from base average: p-value<0.05 (*); p-value<0.01 (**); p-value<0.001 (***).

Supplementary Figure 2 DNA pathogen abundance related to the site, month, and genus. Data are presented log10 transformation for better visualization. Means are visualized are red dots. Different number of asterisks indicate statistical differences from base average: p-value<0.05 (*); p-value<0.01 (**); p-value<0.001 (***).

Supplementary Table 1 Individual pathogen abundance of the collected samples, grouped by genus, species, region, site, and month.

Supplementary Table 4 Wilcoxon test results. Tables show the significativity level of each paired test for each pathogen in different sites, months and genera. Only p-values<0.05 are shown. All the other comparisons (not shown) have p-values>0.05.

Supplementary Table 5 Accession number of viral sequences, deposited in GenBank.

Supplementary Table 6 Associations between wild bee species and honey bee pathogens that were never described before.
