# Supplementary figures and images for: Occurrence of Honey Bee (Apis mellifera L.) Pathogens in Wild Pollinators in Northern Italy
Source: Front Cell Infect Microbiol. 2022 Jun 30;12:907489. doi: 10.3389/fcimb.2022.907489 (PMC9280159; doi:10.3389/fcimb.2022.907489)

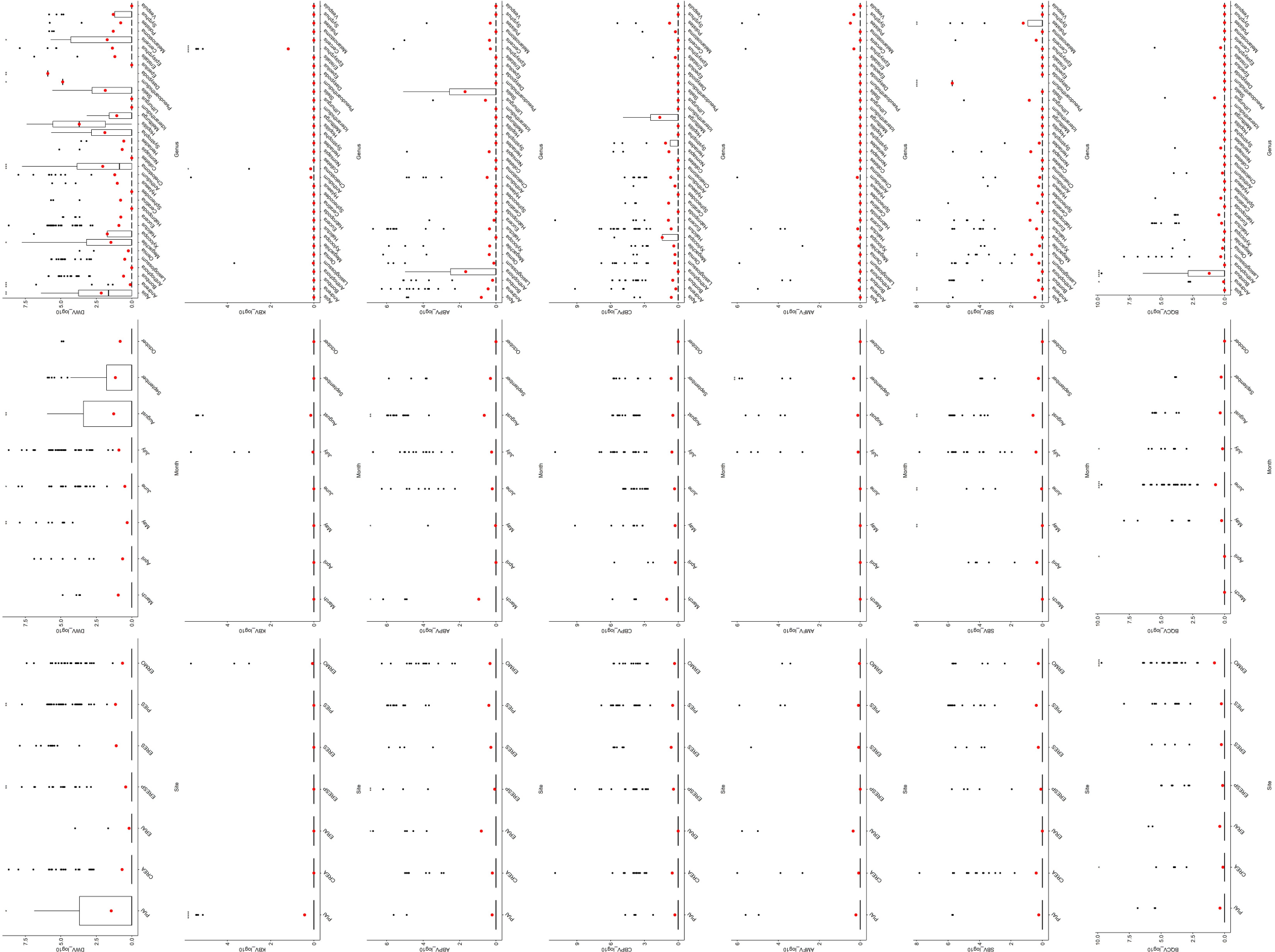

Supplement: Supplementary Figure 1 — Virus abundance related to the site, month, and genus. Data are presented log10 transformation for better visualization. Means are visualized are red dots. Different number of asterisks indicate statistical differences from base average: p-value<0.05 (*); p-value<0.01 (**); p-value<0.001 (***). [file DataSheet_1.pdf]

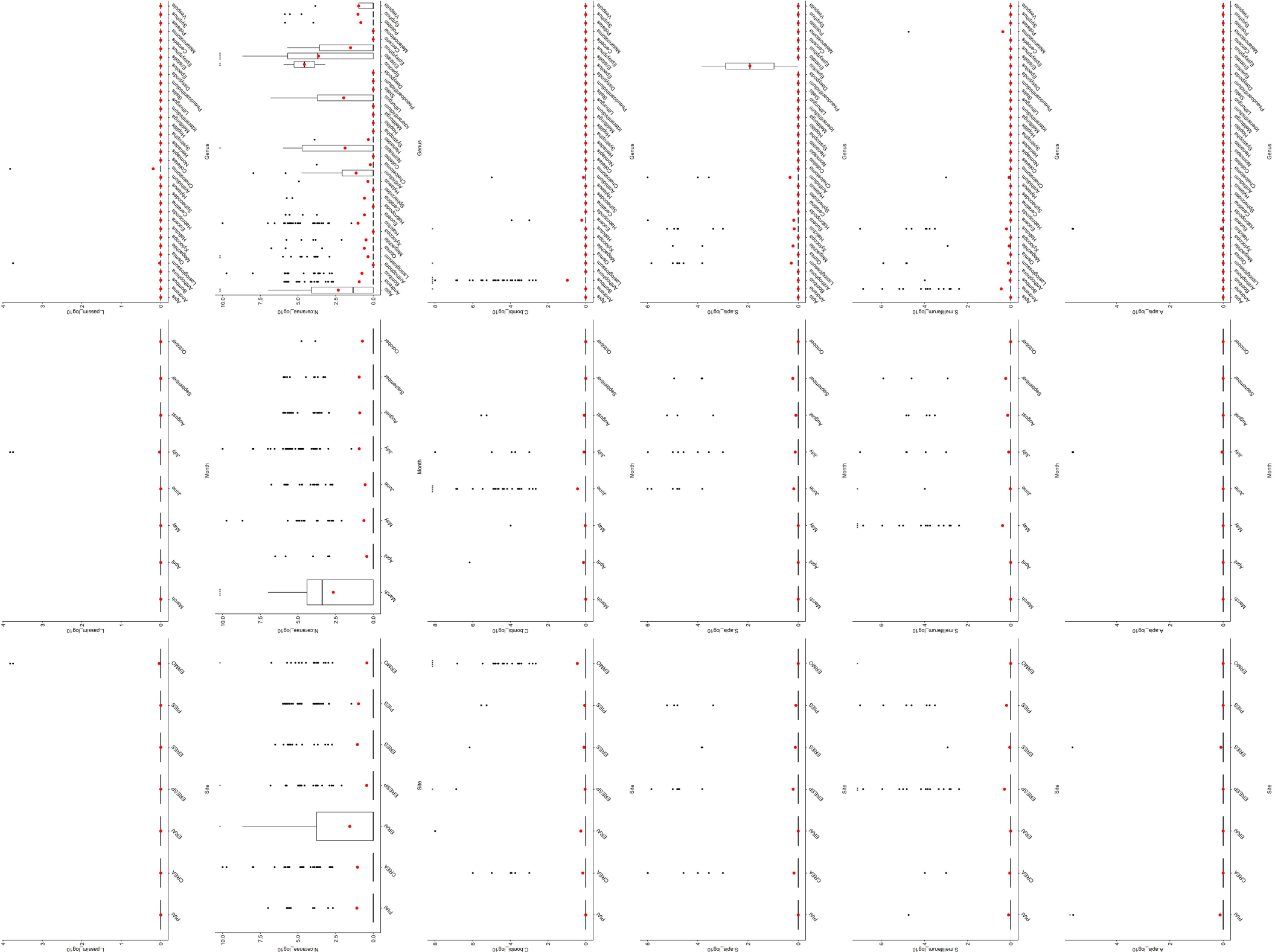

Supplement: Supplementary Figure 2 — DNA pathogen abundance related to the site, month, and genus. Data are presented log10 transformation for better visualization. Means are visualized are red dots. Different number of asterisks indicate statistical differences from base average: p-value<0.05 (*); p-value<0.01 (**); p-value<0.001 (***). [file DataSheet_2.pdf]
