# Supplementary material for: Occurrence of Honey Bee (Apis mellifera L.) Pathogens in Wild Pollinators in Northern Italy
Source: Front Cell Infect Microbiol. 2022 Jun 30;12:907489. doi: 10.3389/fcimb.2022.907489 (PMC9280159; doi:10.3389/fcimb.2022.907489)
Supplement: Supplementary Table 5 — Accession number of viral sequences, deposited in GenBank. [file Table_5.docx]

| **Accession Number** | **Viruses** | **Host** | **Site** |
| --- | --- | --- | --- |
| ON304218 | KBV | *Anthidium loti* | ERMO |
| ON304219 | KBV | *Chelostoma rapunculi* | ERMO |
| ON304220 | KBV | *Lasioglossum pauxillum* | ERMO |
| ON304221 | KBV | *Episyrphus balteatus* | PIAI |
| ON304222 | ABPV | *Anthidium florentinum* | CREA |
| ON304223 | ABPV | *Halictus scabiosae* | CREA |
| ON304224 | ABPV | *Eucera eucnemidea* | CREA |
| ON304225 | ABPV | *Apis mellifera* | CREA |
| ON304226 | ABPV | *Osmia bicornis* | ERESP |
| ON304227 | ABPV | *Andrena lagopus* | ERESP |
| ON304228 | ABPV | *Sphaerophoria scripta* | ERESP |
| ON304229 | ABPV | *Stelis breviscula* | ERESP |
| ON304230 | ABPV | *Lasioglossum pauxillum* | ERAI |
| ON304231 | ABPV | *Andrena impunctata* | ERAI |
| ON304232 | ABPV | *Halictus fulvipes* | ERAI |
| ON304233 | ABPV | *Eucneminae* sp | ERAI |
| ON304234 | ABPV | *Nomiapis diversipes* | ERAI |
| ON304235 | ABPV | *Sceliphron caementarium* | ERAI |
| ON304236 | ABPV | *Polistes* sp | ERAI |
| ON304237 | ABPV | *Andrena flavipes* | ERES |
| ON304238 | ABPV | *Cerceris* sp | ERES |
| ON304239 | ABPV | *Lithurgus cornutus* | ERES |
| ON304240 | ABPV | *Megachile leachella* | ERES |
| ON304241 | ABPV | *Andrena hattorfiana* | ERMO |
| ON304242 | ABPV | *Bombus hortorum* | ERMO |
| ON304243 | ABPV | *Megachile pilidens* | ERMO |
| ON304244 | ABPV | *Anthidium manicatum* | ERMO |
| ON304245 | ABPV | *Osmia aurulenta* | ERMO |
| ON304246 | ABPV | *Bombus humilis appenninus* | ERMO |
| ON304247 | ABPV | *Bombus ruderaius* | ERMO |
| ON304248 | ABPV | *Bombus terrestris* | PIES |
| ON304249 | ABPV | *Bombus pascuorum* | PIES |
| ON304250 | ABPV | *Megachile melanopyga* | PIES |
| ON304251 | ABPV | *Lasioglossum glabriusculum* | PIES |
| ON304252 | ABPV | *Apis mellifera* | PIAI |
| ON448627 | *Am*FV | *Halictus scabiosae* | CREA |
| ON448628 | *Am*FV | *Megachile curcumcinta* | CREA |
| ON448629 | *Am*FV | *Anthidium florentinum* | CREA |
| ON448630 | *Am*FV | *Andrena impunctata* | ERAI |
| ON448631 | *Am*FV | *Polistes* spp | ERAI |
| ON448632 | *Am*FV | *Halictus subauratus* | ERES |
| ON448633 | *Am*FV | *Villa* sp | ERES |
| ON448634 | *Am*FV | *Bombus pratorum* | ERMO |
| ON448635 | *Am*FV | *Bombus rupestris* | ERMO |
| ON448636 | *Am*FV | *Halictus simplex* | PIES |
| ON448637 | *Am*FV | *Halictus scabiosae* | PIES |
| ON448638 | *Am*FV | *Lasioglossum villosulum* | PIES |
| ON448639 | *Am*FV | *Syrphus* sp | PIAI |
| ON448640 | *Am*FV | *Episyrphus balteatus* | PIAI |
| ON448642 | DWV | *Lasioglossum marginatum* | CREA |
| ON448643 | DWV | *Megachile willughbiella* | CREA |
| ON448644 | DWV | *Melanostoma mellinum* | CREA |
| ON448645 | DWV | *Anthidium florentinum* | CREA |
| ON448646 | DWV | *Megachile curcuncinta* | CREA |
| ON448647 | DWV | *Hylaeus* sp | CREA |
| ON448648 | DWV | *Eucera eucnemidea* | CREA |
| ON448649 | DWV | *Halictus scabiosae* | CREA |
| ON448650 | DWV | *Halictus fulvipes* | CREA |
| ON448651 | DWV | *Bombus pascuorum* | CREA |
| ON448652 | DWV | *Halictus simplex* | CREA |
| ON448653 | DWV | *Apis mellifera* | ERESP |
| ON448654 | DWV | *Lasioglossum marginatum* | ERESP |
| ON448655 | DWV | *Scavea pyrastri* | ERESP |
| ON448656 | DWV | *Osmia aurulenta* | ERESP |
| ON448657 | DWV | *Eucera nigrescens* | ERESP |
| ON448658 | DWV | *Eucera clypeata* | ERESP |
| ON448659 | DWV | *Megachile albisecta* | ERESP |
| ON448660 | DWV | *Lasioglossum malachurum* | ERESP |
| ON448661 | DWV | *Halictus scabiosae* | ERESP |
| ON448662 | DWV | *Ceratina cucurbitina* | ERESP |
| ON448663 | DWV | *Lasioglossum corvinum* | ERESP |
| ON448664 | DWV | *Lasioglossum discum* | ERESP |
| ON448665 | DWV | *Ceratina dentiventris* | ERESP |
| ON448666 | DWV | *Stelis breviscula* | ERESP |
| ON448667 | DWV | *Andrena impunctata* | ERAI |
| ON448668 | DWV | *Eucneumidae* sp | ERAI |
| ON448669 | DWV | *Villa* sp | ERAI |
| ON448670 | DWV | *Lasioglossum malachurum* | ERAI |
| ON448671 | DWV | *Megachile melanopyga* | ERES |
| ON448672 | DWV | *Trichodes apiarius* | ERES |
| ON448673 | DWV | *Cerceris sp* | ERES |
| ON448674 | DWV | *Halictus subauratus* | ERES |
| ON448675 | DWV | *Hylaeus gibbus* | ERES |
| ON448676 | DWV | *Systropha curvicornis* | ERES |
| ON448677 | DWV | *Megachile centuncularis* | ERES |
| ON448678 | DWV | *Megachile albisecta* | ERES |
| ON448679 | DWV | *Merodon sp* | ERES |
| ON448680 | DWV | *Bombus sylvestris* | ERMO |
| ON448681 | DWV | *Heriades truncorum* | ERMO |
| ON448682 | DWV | *Bombus lucorum* | ERMO |
| ON448683 | DWV | *Apis mellifera* | ERMO |
| ON448684 | DWV | *Melitturga clavicornis* | ERMO |
| ON448685 | DWV | *Osmia brevicornis* | ERMO |
| ON448686 | DWV | *Xylocopa violacea* | ERMO |
| ON448687 | DWV | *Halictus flavipes* | ERMO |
| ON448688 | DWV | *Bombus sylvarum* | ERMO |
| ON448689 | DWV | *Hoplitis adunca* | ERMO |
| ON448690 | DWV | *Lasioglossum pauxillum* | ERMO |
| ON448691 | DWV | *Heriades rubicola* | ERMO |
| ON448692 | DWV | *Lasioglossum interruptum* | ERMO |
| ON448693 | DWV | *Halictus scabiosae* | ERMO |
| ON448694 | DWV | *Halictus simplex* | ERMO |
| ON448695 | DWV | *Anthidium florentinum* | ERMO |
| ON448696 | DWV | *Halictus fulvipes* | ERMO |
| ON448697 | DWV | *Lasioglossum villosulum* | ERMO |
| ON448698 | DWV | *Bombus pascuorum* | ERMO |
| ON448699 | DWV | *Hylaeus angustatus* | ERMO |
| ON448700 | DWV | *Bombus lapidarius* | ERMO |
| ON448701 | DWV | *Bombus rupestris* | ERMO |
| ON448702 | DWV | *Lasioglossum marginatum* | PIES |
| ON448703 | DWV | *Chelostoma rapunculi* | PIES |
| ON448704 | DWV | *Chelostoma campanularum* | PIES |
| ON448705 | DWV | *Sphaerophoria scripta* | PIES |
| ON448706 | DWV | *Nomiapis diversipes* | PIES |
| ON448707 | DWV | *Melostoma mellinum* | PIES |
| ON448708 | DWV | *Megachile pilidens* | PIES |
| ON448709 | DWV | *Lasioglossum malachurum* | PIES |
| ON448710 | DWV | *Halictus scabiosae* | PIES |
| ON448711 | DWV | *Polistes dominula* | PIES |
| ON448712 | DWV | *Lasioglossum villosulum* | PIES |
| ON448713 | DWV | *Bombus terrestris* | PIES |
| ON448714 | DWV | *Bombus pascuorum* | PIES |
| ON448715 | DWV | *Dasypoda hirtipes* | PIES |
| ON448716 | DWV | *Episyrphus balteatus* | PIES |
| ON448717 | DWV | *Syrphus ribesii* | PIES |
| ON448718 | DWV | *Apis mellifera* | PIAI |
| ON448719 | DWV | *Eristalis tenax* | PIAI |
| ON448720 | DWV | *Halictus subauratus* | PIAI |
| ON448721 | DWV | *Cerceris sp* | PIAI |
| ON448722 | DWV | *Nomiapis diversipes* | PIAI |
| ON448723 | DWV | *Halictus simplex* | PIAI |
| ON448724 | DWV | *Ceratina gravidula* | PIAI |
| ON448725 | DWV | *Episyrphus baltealtus* | PIAI |
| ON448726 | DWV | *Pseudoanthidium scapulare* | PIAI |
| ON448727 | DWV | *Syrphus sp* | PIAI |
| ON448728 | SBV | *Osmia aurulenta* | CREA |
| ON448729 | SBV | *Osmia caerulescens* | CREA |
| ON448730 | SBV | *Osmia rufohirta* | CREA |
| ON448731 | SBV | *Eucera eucnemidea* | CREA |
| ON448732 | SBV | *Anthidium florentinum* | CREA |
| ON448733 | SBV | *Halictus fulvipes* | CREA |
| ON448734 | SBV | *Lasioglossum discum* | CREA |
| ON448735 | SBV | *Apis mellifera* | CREA |
| ON448736 | SBV | *Lithurgus cornutus* | ERESP |
| ON448737 | SBV | *Halictus scabiosae* | ERESP |
| ON448738 | SBV | *Lasioglossum malachurum* | ERESP |
| ON448739 | SBV | *Lasioglossum corvinum* | ERESP |
| ON448740 | SBV | *Eucneumidae* | ERAI |
| ON448741 | SBV | *Lasioglossum clypeare* | ERES |
| ON448742 | SBV | *Cerceris sp* | ERES |
| ON448743 | SBV | *Megachile centuncularis* | ERES |
| ON448744 | SBV | *Nomiapis diversipes* | ERES |
| ON448745 | SBV | *Heriades rubicola* | ERMO |
| ON448746 | SBV | *Lasioglossum malachurum* | ERMO |
| ON448747 | SBV | *Sphaerophoria sp* | ERMO |
| ON448748 | SBV | *Bombus humilis appenninus* | ERMO |
| ON448749 | SBV | *Halictus simplex* | ERMO |
| ON448750 | SBV | *Halictus scabiosae* | ERMO |
| ON448751 | SBV | *Halictus fulvipes* | ERMO |
| ON448752 | SBV | *Bombus lucorum* | ERMO |
| ON448753 | SBV | *Hylaeus angustatus* | ERMO |
| ON448754 | SBV | *Bombus lapidarius* | ERMO |
| ON448755 | SBV | *Nomiapis_diversipes* | PIAI |
| ON448756 | SBV | *Pseudanthidium scapulare* | PIAI |
| ON448757 | SBV | *Ceratina dentiventris* | PIES |
| ON448758 | SBV | *Polistes dominula* | PIES |
| ON448759 | SBV | *Lasioglossum villosulum* | PIES |
| ON448760 | SBV | *Bombylius sp* | PIES |
| ON448761 | SBV | *Bombus pascuorum* | PIES |
| ON448762 | SBV | *Halictus scabiosae* | PIES |
| ON448763 | SBV | *Halictus fulvipes* | PIES |
| ON448764 | SBV | *Megachile melanopyga* | PIES |
| ON448765 | BQCV | *Halictus scabiosae* | CREA |
| ON448766 | BQCV | *Eucera eucnemidea* | CREA |
| ON448767 | BQCV | *Anthidium florentinum* | CREA |
| ON448768 | BQCV | *Halictus simplex* | CREA |
| ON448769 | BQCV | *Andrena distinguenda* | ERESP |
| ON448770 | BQCV | *Lasioglossum politum* | ERESP |
| ON448771 | BQCV | *Megachile ericetorum* | ERESP |
| ON448772 | BQCV | *Osmia caerulescens* | ERESP |
| ON448773 | BQCV | *Lasioglossum marginatum* | ERESP |
| ON448774 | BQCV | *Halictus scabiosae* | ERESP |
| ON448775 | BQCV | *Bombus pascuorum* | ERMO |
| ON448776 | BQCV | *Bombus sylvestris* | ERMO |
| ON448777 | BQCV | *Bombus ruderatus* | ERMO |
| ON448778 | BQCV | *Bombus lapidarius* | ERMO |
| ON448779 | BQCV | *Bombus lucorum* | ERMO |
| ON448780 | BQCV | *Bombus campestris* | ERMO |
| ON448781 | BQCV | *Bombus rupestris* | ERMO |
| ON448782 | BQCV | *Andrena ventricosa* | ERES |
| ON448783 | BQCV | *Lithurgus cornutus* | ERES |
| ON448784 | BQCV | *Halictus subauratus* | ERES |
| ON448785 | BQCV | *Merodon sp* | ERES |
| ON448786 | BQCV | *Nomiapis diversipes* | ERES |
| ON448787 | BQCV | *Lasioglossum malachurum* | ERAI |
| ON448788 | BQCV | *Halictus simplex* | ERAI |
| ON448789 | BQCV | *Eucera nigrifacies* | PIES |
| ON448790 | BQCV | *Andrena ventricosa* | PIES |
| ON448791 | BQCV | *Andrena humilis* | PIES |
| ON448792 | BQCV | *Halictus scabiosae* | PIES |
| ON448793 | BQCV | *Bombus pascuorum* | PIES |
| ON448794 | BQCV | *Bombylius sp* | PIES |
| ON448795 | BQCV | *Lasioglossum malachurum* | PIES |
| ON448796 | BQCV | *Lasioglossum peregrinum* | PIAI |
| ON448797 | BQCV | *Ceratina gravidula* | PIAI |
| ON448798 | BQCV | *Episyrphus balteatus* | PIAI |
| ON448799 | CBPV | *Osmia aurulenta* | CREA |
| ON448800 | CBPV | *Lasioglossum glabriusculum* | CREA |
| ON448801 | CBPV | *Bombus pascuorum* | CREA |
| ON448802 | CBPV | *Anthidium florentinum* | CREA |
| ON448803 | CBPV | *Halictus fulvipes* | CREA |
| ON448804 | CBPV | *Eucera eucnemidea* | CREA |
| ON448805 | CBPV | *Lasioglossum politum* | CREA |
| ON448806 | CBPV | *Lasioglossum marginatum* | ERESP |
| ON448807 | CBPV | *Andrena lagopus* | ERESP |
| ON448808 | CBPV | *Andrena nitida* | ERESP |
| ON448809 | CBPV | *Melanostoma sp* | ERESP |
| ON448810 | CBPV | *Eucera clypeata* | ERESP |
| ON448811 | CBPV | *Bombus terrestris* | ERESP |
| ON448812 | CBPV | *Bombus lapidarius* | ERESP |
| ON448813 | CBPV | *Bombus pascuorum* | ERESP |
| ON448814 | CBPV | *Megachile ericetorum* | ERESP |
| ON448815 | CBPV | *Halictus subauratus* | ERESP |
| ON448816 | CBPV | *Megachile albisecta* | ERESP |
| ON448817 | CBPV | *Halictus scabiosae* | ERESP |
| ON448818 | CBPV | *Ceratina chalybea* | ERESP |
| ON448819 | CBPV | *Eucera eucnemidea* | ERESP |
| ON448820 | CBPV | *Halictus simplex* | ERESP |
| ON448821 | CBPV | *Ceratina chalcites* | ERESP |
| ON448822 | CBPV | *Villa sp* | ERAI |
| ON448823 | CBPV | *Xylocopa valga* | ERES |
| ON448824 | CBPV | *Andrena ferrugineicrus* | ERES |
| ON448825 | CBPV | *Andrena impunctata* | ERES |
| ON448826 | CBPV | *Halictus cochlearitarsis* | ERES |
| ON448827 | CBPV | *Volucella zonaria* | ERES |
| ON448828 | CBPV | *Lasioglossum malachurum* | ERES |
| ON448829 | CBPV | *Heriades rubicola* | ERES |
| ON448830 | CBPV | *Heriades crenulata* | ERES |
| ON448831 | CBPV | *Villa sp* | ERES |
| ON448832 | CBPV | *Nomiapis diversipes* | ERES |
| ON448833 | CBPV | *Megachile melanopyga* | ERMO |
| ON448834 | CBPV | *Apis mellifera* | ERMO |
| ON448835 | CBPV | *Melitturga clavicornis* | ERMO |
| ON448836 | CBPV | *Megachile leachella* | ERMO |
| ON448837 | CBPV | *Heriades rubicola* | ERMO |
| ON448838 | CBPV | *Osmia aurolenta* | ERMO |
| ON448839 | CBPV | *Halictus simplex* | ERMO |
| ON448840 | CBPV | *Halictus fulvipes* | ERMO |
| ON448841 | CBPV | *Hylaeus punctatus* | ERMO |
| ON448842 | CBPV | *Odynerus sp* | ERMO |
| ON448843 | CBPV | *Bombus lapidarius* | ERMO |
| ON448844 | CBPV | *Bombus pascuorum* | ERMO |
| ON448845 | CBPV | *Bombus rupestris* | ERMO |
| ON448846 | CBPV | *Bombus pratorum* | ERMO |
| ON448847 | CBPV | *Apis mellifera* | PIES |
| ON448848 | CBPV | *Bombus vestalis* | PIES |
| ON448849 | CBPV | *Eristalis tenax* | PIES |
| ON448850 | CBPV | *Ceratina gravidula* | PIES |
| ON448851 | CBPV | *Nomiapis diversipes* | PIAI |
| ON448852 | CBPV | *Lasioglossum leucozonium* | PIAI |
| ON448853 | CBPV | *Lasioglossum discum* | PIAI |
| ON448854 | CBPV | *Halictus simplex* | PIAI |
| ON448855 | CBPV | *Ceratina cucurbitina* | PIAI |
| ON448856 | CBPV | *Halictus scabiosae* | PIAI |
| ON448857 | CBPV | *Polistes dominula* | PIAI |
| ON448858 | CBPV | *Bombus pascuorum* | PIAI |
| ON448859 | CBPV | *Lasioglossum malachurum* | PIAI |
| ON448860 | CBPV | *Bombus terrestris* | PIAI |
| ON448861 | CBPV | *Lasioglossum villosulum* | PIAI |
| ON448862 | CBPV | *Halictus fulvipes* | PIAI |
| ON448763 | CBPV | *Scavea pyrastri* | PIAI |
