# Supplementary material for: Occurrence of Honey Bee (Apis mellifera L.) Pathogens in Wild Pollinators in Northern Italy
Source: Front Cell Infect Microbiol. 2022 Jun 30;12:907489. doi: 10.3389/fcimb.2022.907489 (PMC9280159; doi:10.3389/fcimb.2022.907489)
Supplement: Supplementary Table 6 — Associations between wild bee species and honey bee pathogens that were never described before. [file Table_6.docx]

| **Host species** | **Honey bee pathogens detected** |
| --- | --- |
| *Andrena agilissima* | *N. ceranae* |
| *Andrena decipens* | *N. ceranae* |
| *Andrena distinguenda* | BQCV, *N. ceranae, S. melliferum* |
| *Andrena ferruginecruis* | CBPV |
| *Andrena flavipes* | DWV, *N. ceranae* |
| *Andrena hattorfiana* | ABPV |
| *Andrena humilis* | *N. ceranae* |
| *Andrena impunctata* | ABPV, CBPV, AmFV, N*. ceranae, S. melliferum* |
| *Andrena lagopus* | ABPV, CBPV, *N. ceranae, S. melliferum* |
| *Andrena minutula* | *N. ceranae* |
| *Andrena minutuloides* | *N. ceranae* |
| *Andrena nitida* | ABPV |
| *Andrena nitiduscula* | *N. ceranae* |
| *Andrena ovatula* | *N. ceranae* |
| *Andrena proxima* | *N. ceranae* |
| *Andrena ventricosa* | BQCV |
| *Andrena viridescens* | *N. ceranae, S. mellifeerum* |
| *Anthidium loti* | KBV |
| *Bombus campestris* | BQCV |
| *Bombus sylvestris* | DWV |
| *Ceratina chalcites* | CBPV |
| *Ceratina chalybea* | CBPV |
| *Ceratina cucurbitina* | DWV, CBPV |
| *Ceratina dentiventris* | DWV, SBV, *N. ceranae* |
| *Ceratina gravidula* | DWV, CBPV, BQCV |
| *Chelostoma campanularum* | DWV |
| *Chelostoma rapunculi* | DWV, KBV, *N. ceranae, L. passim* |
| *Dasypoda hirtipes* | DWV |
| *Epeolus cruciger* | *N. ceranae, S. apis* |
| *Epeolus variegatus* | *N. ceranae* |
| *Eucera clypeata* | DWV, CBPV |
| *Eucera eucnemidea* | DWV, ABPV, SBV, BQCV, *N. ceranae, C. bombi, S. apis* |
| *Eucera nigrifacies* | BQCV |
| *Halictus scabiosae* | DWV, KBV, ABPV, CBPV, AmFV, SBV, BQCV, *N. ceranae, S. apis, S. melliferum* |
| *Halictus subauratus* | DWV, CBPV, AmFV, BQCV, *N. ceranae* |
| *Halictus cochlearitarsis* | CBPV, *A. apis* |
| *Heriades crenulata* | CBPV, *N. ceranae* |
| *Heriades rubicula* | DWV, ABPV, CBPV, SBV |
| *Hyaleus gibbus* | DWV, *N. ceranae* |
| *Hylaeus punctatus* | CBPV |
| *Hylaeus angustatus* | DWV, SBV |
| *Lasioglossum clypeare* | SBV |
| *Lasioglossum corvinum* | DWV, SBV, *N. ceranae* |
| *Lasioglossum discum* | DWV, CBPV, SBV, *N. ceranae* |
| *Lasioglossum glabriusculum* | ABPV, CBPV |
| *Lasioglossum interruptum* | DWV, *S. apis* |
| *Lasioglossum leucozonium* | CBPV |
| *Lasioglossum marginatum* | DWV, CBPV, BQCV, *N. ceranae* |
| *Lasioglossum minutissimum* | *N. ceranae* |
| *Lasioglossum peregrinum* | BQCV |
| *Lasioglossum politum* | CBPV, BQCV, *S. apis* |
| *Lasioglossum punctatissimum* | *N. ceranae* |
| *Lasioglossum villosulum* | DWV, ABPV, AmFV, SBV, *N. ceranae, S. melliferum,* |
| *Lithurgus cornutus* | BQCV, *N. ceranae* |
| *Megachile centuncularis* | DWV, SBV |
| *Megachile circumcincta* | DWV, AmFV |
| *Megachile ericetorum* | CBPV, BQCV |
| *Megachile leachella* | ABPV, CBPV |
| *Megachile melanopyga* | DWV, ABPV, CBPV, SBV |
| *Megachile pilidens* | DWV |
| *Megachile pilidens* | ABPV |
| *Megachile willughbiella* | DWV, CBPV, *N. ceranae* |
| *Melitturga clavicornis* | DWV, CBPV |
| *Nomiapis diversipes* | DWV, ABPV, CBPV, SBV, BQCV, *N. ceranae* |
| *Osmia aurulenta* | DWV, ABPV, CBPV, SBV, *N. ceranae* |
| *Osmia brevicornis* | DWV |
| *Osmia caerulescens* | SBV, BQCV |
| *Osmia rufohirta* | SBV, *N. ceranae* |
| *Pseudoanthidium scapulare* | DWV, SBV |
| *Stelis breviuscula* | DWV, ABPV |
| *Systropha curvicornis* | DWV |
| *Xylocopa valga* | CBPV |
